# Human Subtilase SKI-1/S1P Is a Master Regulator of the HCV Lifecycle and a Potential Host Cell Target for Developing Indirect-Acting Antiviral Agents

**DOI:** 10.1371/journal.ppat.1002468

**Published:** 2012-01-05

**Authors:** Andrea D. Olmstead, Wolfgang Knecht, Ina Lazarov, Surjit B. Dixit, François Jean

**Affiliations:** 1 Department of Microbiology and Immunology, Life Sciences Centre, University of British Columbia, Vancouver, British Columbia, Canada; 2 Lead Generation - Target Production, AstraZeneca R&D Mölndal, Mölndal, Sweden; 3 Zymeworks, Vancouver, British Columbia, Canada; University of Kentucky College of Medicine, United States of America

## Abstract

HCV infection is a major risk factor for liver cancer and liver transplantation worldwide. Overstimulation of host lipid metabolism in the liver by HCV-encoded proteins during viral infection creates a favorable environment for virus propagation and pathogenesis. In this study, we hypothesize that targeting cellular enzymes acting as master regulators of lipid homeostasis could represent a powerful approach to developing a novel class of broad-spectrum antivirals against infection associated with human *Flaviviridae* viruses such as hepatitis C virus (HCV), whose assembly and pathogenesis depend on interaction with lipid droplets (LDs). One such master regulator of cholesterol metabolic pathways is the host subtilisin/kexin-isozyme-1 (SKI-1) – or site-1 protease (S1P). SKI-1/S1P plays a critical role in the proteolytic activation of sterol regulatory element binding proteins (SREBPs), which control expression of the key enzymes of cholesterol and fatty-acid biosynthesis. Here we report the development of a SKI-1/S1P-specific protein-based inhibitor and its application to blocking the SREBP signaling cascade. We demonstrate that SKI-1/S1P inhibition effectively blocks HCV from establishing infection in hepatoma cells. The inhibitory mechanism is associated with a dramatic reduction in the abundance of neutral lipids, LDs, and the LD marker: adipose differentiation-related protein (ADRP)/perilipin 2. Reduction of LD formation inhibits virus assembly from infected cells. Importantly, we confirm that SKI-1/S1P is a key host factor for HCV infection by using a specific active, site-directed, small-molecule inhibitor of SKI-1/S1P: PF-429242. Our studies identify SKI-1/S1P as both a novel regulator of the HCV lifecycle and as a potential host-directed therapeutic target against HCV infection and liver steatosis. With identification of an increasing number of human viruses that use host LDs for infection, our results suggest that SKI-1/S1P inhibitors may allow development of novel broad-spectrum biopharmaceuticals that could lead to novel indirect-acting antiviral options with the current standard of care.

## Author Summary

Chronic hepatitis C virus (HCV) infection is one of the leading causes of liver cancer and liver transplantation worldwide. No vaccine is available for preventing the spread of HCV, and the current therapeutic regimen is only moderately effective and causes serious side effects. New antiviral agents are required to treat HCV infection, but the high mutation rate of HCV hinders the effectiveness of virus-specific inhibitors. Targeting the host enzymes required for HCV to replicate offers a promising new direction for antiviral therapy. During infection, HCV promotes excessive fat accumulation in the liver, which benefits the virus as this promotes formation of lipid droplets, a cellular organelle essential for assembly of new HCV infectious viral particles. Here, we report the development of a specific inhibitor targeting SKI-1/S1P, a host enzyme required for lipid production in human cells. We show that inhibiting SKI-1/S1P activity in human liver cells effectively blocks lipid droplet formation and HCV infection. Many prevalent human viruses, such as dengue, rotavirus, and hepatitis B virus, hijack host lipid metabolic pathways similar to those targeted by HCV to complete their lifecycle. Thus, we propose that cellular SKI-1/S1P is a potential target for developing desperately needed novel broad-spectrum antiviral drugs.

## Introduction

Hijacking of host lipids and their biosynthetic pathways is a common strategy for microbial infection. Human enveloped viruses including hepatitis C virus (HCV) and human immunodeficiency virus (HIV)-1 use cholesterol-rich lipid rafts for entry [1], [2], assembly [3], and/or replication [2], [4]. Lipid droplets (LDs), once considered static storage vesicles for host lipids, are now appreciated as dynamic organelles [5] that are also utilized in the lifecycles of pathogenic human viruses including rotavirus (RV) [6], dengue virus (DV) [7], and HCV [8]. HCV in particular requires host LDs for assembly of nascent viral particles [9]-[11].

HCV is a globally important human pathogen afflicting more than 170 million people worldwide [12], [13]. HCV, a hepacivirus member of the *Flaviviridae* family and an enveloped virus, is encoded by a single-stranded positive-sense RNA genome [14]. Viral RNA is directly translated by the host machinery into a single polyprotein, which is cleaved by host and virus-encoded proteases to release the individual structural (core, E1, and E2) and non-structural (NS) proteins (p7, NS2, NS3, NS4A, NS4B, NS5A, and NS5B) [15]. During infection, HCV-encoded proteins promote reorganization and accumulation of LDs in the perinuclear region of the cell [16]. The HCV core protein is targeted to LDs [17] and orchestrates the assembly and release of infectious viral particles during the late stages of infection [18]. Hence, disrupting the interaction of the HCV core protein with LDs compromises this essential stage within the HCV lifecycle [8], [10], [11].

Several host metabolic pathways tightly control cellular lipid synthesis. Targeted disruption of these pathways [19]-[21] by HCV-encoded proteins has been linked with liver steatosis [22], [23] in HCV-infected individuals. Importantly, there is a correlation between the degree of steatosis and both the severity of chronic HCV infection [13], [24] and the response to treatment with pegylated-interferon-α and ribavirin [25], [26].

Overstimulation of host lipid metabolism by HCV during infection is achieved through a variety of molecular mechanisms (reviewed in [27], [28], and [29]). For example, HCV employs multiple strategies to activate the sterol regulatory element binding protein (SREBP) pathway, which is important for regulation of host lipid homeostasis [19], [30]–[32]. SREBPs are endoplasmic reticulum (ER), membrane-anchored transcription factors that respond to changes in intracellular sterol levels through interactions with sterol-sensing proteins (reviewed in [33]). When sterol levels are high, SREBPs are retained as inactive precursors in the ER [34], [35]. Under low sterol conditions, SREBPs are escorted to the Golgi, where two resident endoproteases (subtilisin kexin isozyme/site-1 protease (SKI-1/S1P) and SREBP Site-2 protease (S2P); reviewed in [36] and see below) cleave the precursor polypeptide SREBP, allowing the release of transcriptionally active SREBP molecules from the ER [37], [38]. The released SREBP fragment migrates to the nucleus and binds to sterol response elements in the promoters of cholesterol and fatty acid biosynthetic target genes, and it activates their transcription [39]–[41]. Activation of the SREBP pathway by HCV aids the virus lifecycle and may ultimately promote the development of steatosis and liver disease in chronically infected individuals [42], [43].

Human site-1 protease (S1P, MEROPS S08.8063), also widely known as subtilisin/kexin-isozyme-1 (SKI-1), is a membrane-bound subtilisin-related serine endoprotease that belongs to a group of nine mammalian proprotein convertases (PCs) in family S08 [44]–[46]. SKI-1/S1P displays unique substrate specificity among the PC members by showing preferred cleavage after non-basic amino acids [47]. SKI-1/S1P cleaves at the carboxy-terminus of the peptidyl sequence Arg/Lys-Xaa_3_-Xaa_2_-Leu/Ser/Thr [48], where Xaa_3_ is any amino acid except Cys at the P3 position of the scissile peptide bond and Xaa_2_ is a hydrophobic amino acid containing an alkyl side chain at the P2 position [49]. In addition to the proteolytic processing of transcription factors [36], SKI-1/S1P participates in the proteolytic activation of viral-envelope glycoproteins of the Lassa virus [50], the lymphocytic choriomeningitis virus [51], and the Crimean Congo hemorrhagic fever virus [52]. Importantly, endoproteolytic processing of these viral glycoproteins by SKI-1/S1P is a critical step for the production of infectious progeny viruses, suggesting that SKI-1/S1P may represent an attractive target for therapeutic intervention against human pathogenic arenaviruses [47], [48], [50]–[52].

Given that SKI-1/S1P-dependent proteolytic cleavage of SREBPs is a master molecular switch for controlling host cell cholesterol homeostasis [41], [53], we hypothesized that inhibiting SKI-1/S1P endoproteolytic activity in the secretory pathway would prevent HCV hijacking of host lipid metabolic pathways and thus compromise the virus lifecycle. Because of our previous success with engineering serine protease inhibitors (serpins) to develop effective and selective PC inhibitors [54], [55], we hypothesized that engineering a naturally occurring serpin scaffold could also provide a powerful approach for developing selective SKI-1/S1P inhibitors. Serpins differ from non-serpin inhibitors in that they require a large inhibitor conformational change in order to trap proteases in an irreversible complex [56]. The conformational change is initiated by reaction of the active serine of the protease with the reactive center loop (RCL) of the serpin, which results in a covalent species involving an acyl ester linkage to the γO of the protease active site serine [57]. This cleaves the RCL, which then moves 71 Å to the opposite pole of the serpin, taking the tethered protease with it [57].

We selected a *Drosophila* serpin, Spn4A, as a prototype macromolecular inhibitor scaffold. The identification of Spn4A by our group as the most potent natural serpin inhibitor of the human PC furin (K_i_,13 pM [58]) provides us with a novel and unique molecular tool for dissecting the contribution of SKI-1/S1P-dependent proteolytic activity in the secretory pathway to viral infection of eukaryotic cells. We report that engineering of the RCL of Spn4A to mimic the consensus sequence Arg/Lys^P4^-Xaa_3_-Xaa_2_-Leu/Ser/Thr^P1↓^ for cleavage by SKI-1/S1P (Spn4A RCL: Arg^P4^-Arg-Lys-Arg ^P1↓^ -> Arg^P4^-Arg-Leu-Leu ^P1↓^) resulted in the development of Spn4A-RRLL, a novel, selective, and effective serpin-based inhibitor of SKI-1/S1P. We demonstrated the anti-proteolytic and anti-HCV activities of our new recombinant adenovirus-expressing Spn4A-RRLL “secreted” (s) variant directed at the secretory pathway SKI-1/S1P. Expression of Spn4A.RRLL(s) in Huh-7.5.1 cells results in a strong inhibition of the SKI-1/S1P-mediated activation of SREBP-1 and down-regulation of SREBP target gene products. As hypothesized, inhibiting SKI-1/S1P activity robustly blocked HCV infection of Huh-7.5.1 cells in a dose-dependent manner. We found that specific inhibition of SKI-1/S1P activity by Spn4A.RRLL(s) dramatically reduced the abundance of LDs in hepatoma cells. Use of the specific active site-directed small-molecule inhibitor of SKI-1/S1P, PF-429242, confirmed the results of our studies with the protein-based therapeutic Spn4A.RRLL(s), with a robust inhibition of HCV infection.

The results of our studies contribute to our understanding of the HCV lifecycle and HCV-associated steatogenesis and to efforts in developing novel host-directed antiviral therapeutic agents against HCV. In addition, with the finding that an increasing number of human enveloped viruses employ host LDs for infection [6], [7], our results suggest that SKI-1/S1P-directed inhibitors may allow the development of novel broad-spectrum antiviral agents.

## Results

### Protein engineering of the Spn4A scaffold and drug delivery strategy to target secretory pathway SKI-1/S1P

**Figure 1:**
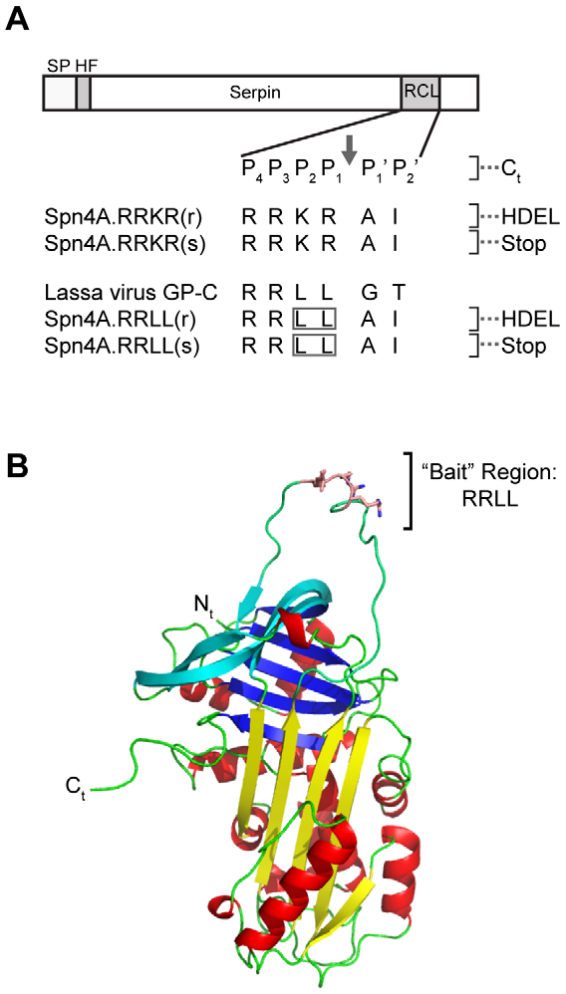
Engineering the Spn4A scaffold to target the human subtilase SKI-1/S1P. (A) Spn4A variants and amino-acid sequences of the engineered reactive center loop (RCL) “bait” region are shown. Spn4A.RRKR(r) encodes for the naturally occurring serpin Spn4A, isolated from *Drosophila melanogaster*, with potent inhibitory activity against the human proprotein convertase furin. Spn4A.RRKR(r) contains the alpha-1 antitrypsin signal peptide (SP) at the N-terminus followed by a tandem His-tag (HHHHHH) and FLAG-tag (DYKDDDDK) sequence (HF). The P4 – P1 furin cleavage sequence in the RCL is Arg-Arg-Lys-Arg. Spn4A.RRKR(r) also contains the His-Asp-Glu-Leu (HDEL) ER retention motif (r) at the C-terminus. The secreted (s) serpin, Spn4A.RRKR(s), contains a stop codon before the HDEL signal. The RCL of Spn4A-RRKR(r) and (s) was modified to mimic the predicted SKI-1/S1P target cleavage site present in the Lassa virus glycoprotein pre-GP-C, which is Arg-Arg-Leu-Leu. Thus, Spn4A.RRLL(r), which is also retained in the ER, encodes the P4 – P1 Arg-Arg-Leu-Leu cleavage recognition sequence in the RCL. Spn4A.RRLL(s) contains a stop codon before HDEL, allowing the serpin to be secreted. (B) *In silico* homology model of the Spn4A.RRLL(r) variant was generated as described in the Materials and Methods. Ribbon diagram of the molecular model was generated using Pymol. The side chains of the RRLL residues within the flexible “bait region” of the RCL are shown as sticks in wheat colour. Sheet A is shown in yellow, sheet B is in blue, and sheet C is in cyan. Alpha-helices are red and loops are green.

Our previous studies have demonstrated that Spn4A architecture can inhibit two evolutionary divergent members of the PC family (furin and PC2) [58]. We selected this novel *Drosophila melanogaster* serpin scaffold to engineer a novel protein-based inhibitor directed at the PC SKI-1/S1P. First, we cloned our pre-His/FLAG-tagged Spn4A construct [58] into an adenoviral shuttle vector to generate Spn4A.RRKR(r) (Figure 1A). Spn4A is a unique “retained” (r) serpin that presents, at its C-terminus, an HDEL sequence (Figure 1A), a functional variant of the C-terminal KDEL retention signal that directs secretory protein retention in the ER [59]. Because SKI-1/S1P cleavage of SREBP substrates occurs in the Golgi apparatus [35], we next needed to generate a “secreted” (s) variant of Spn4A, Spn4A.RRKR(s) (Figure 1A). We hypothesized that only an Spn4A (s) variant, which traffics through the late secretory pathway prior to secretion in the extracellular space, would encounter active SKI-1/S1P molecules in the early Golgi compartment. This was accomplished by inserting a stop codon before the C-terminal ER-retention signal, HDEL. Next, we employed site-directed mutagenesis to optimize the interactions between the RCL of Spn4A and the substrate binding sites of SKI-1/S1P. Residues at positions P2 and P1 of the Spn4A RCL “bait” region Arg^P4^-Arg^P3^-Lys ^P2^-Arg ^P1↓^ were substituted to generate Arg^P4^-Arg^P3^-Leu ^P2^-Leu ^P1↓^, thereby mimicking the Lassa virus glycoprotein precursor GP-C cleavage site [50] (Figure 1A and 1B: Spn4A.RRLL(r) and Spn4A.RRLL(s), respectively).

To test the serpin-like properties and antiviral activities of our Spn4A variants *in cellulo*, recombinant adenoviruses (Ad) expressing the Spn4A constructs Ad-Spn4A.RRLL(r) and Ad-Spn4A.RRLL(s) were produced as described previously [55]. As adenoviruses display strong tropism for the liver [60], the major site of HCV infection [13], these recombinant adenoviruses are especially useful molecular tools for HCV research, including the use of human hepatoma Huh-7.5.1 cells, which support robust HCV infection *in cellulo* [61].

### Robust intracellular expression and differential secretion of adenovirus-expressed Spn4A.RRLL(r) and Spn4A.RRLL(s) in human hepatoma Huh-7.5.1 cells

The level of expression of Spn4A variants was first optimized by infecting human hepatoma Huh-7.5.1 cells (highly permissive for HCV JFH-1 infection) with different multiplicity of infection (moi) of recombinant adenovirus expressing intracellularly retained Spn4A.RRLL(r). Cellomics HCS analysis revealed that over 90% of these Huh-7.5.1 cells expressed Spn4A.RRLL(r) at a moi of 50 (Figure S1). Cellomics HCS was also used to determine if cell death occurs following 2 days of pre-treatment with Spn4A.RRLL(r) or Spn4A.RRLL(s) compared to the control (Ad-Empty) followed by 72 hours of HCV infection as employed in the experiments below (Figure S2). We observed no significant reductions in total cell numbers under these experimental conditions. The high-content analysis of cell death was confirmed using an MTS-based cell viability assay (data not shown). The lack of detectable toxicity induced by Ad-Spn4A.RRLL(r) and Ad-Spn4A.RRLL(s) up to a moi of 50 showed that these variants can be tested over a very wide dynamic range in hepatoma cells.

**Figure 2:**
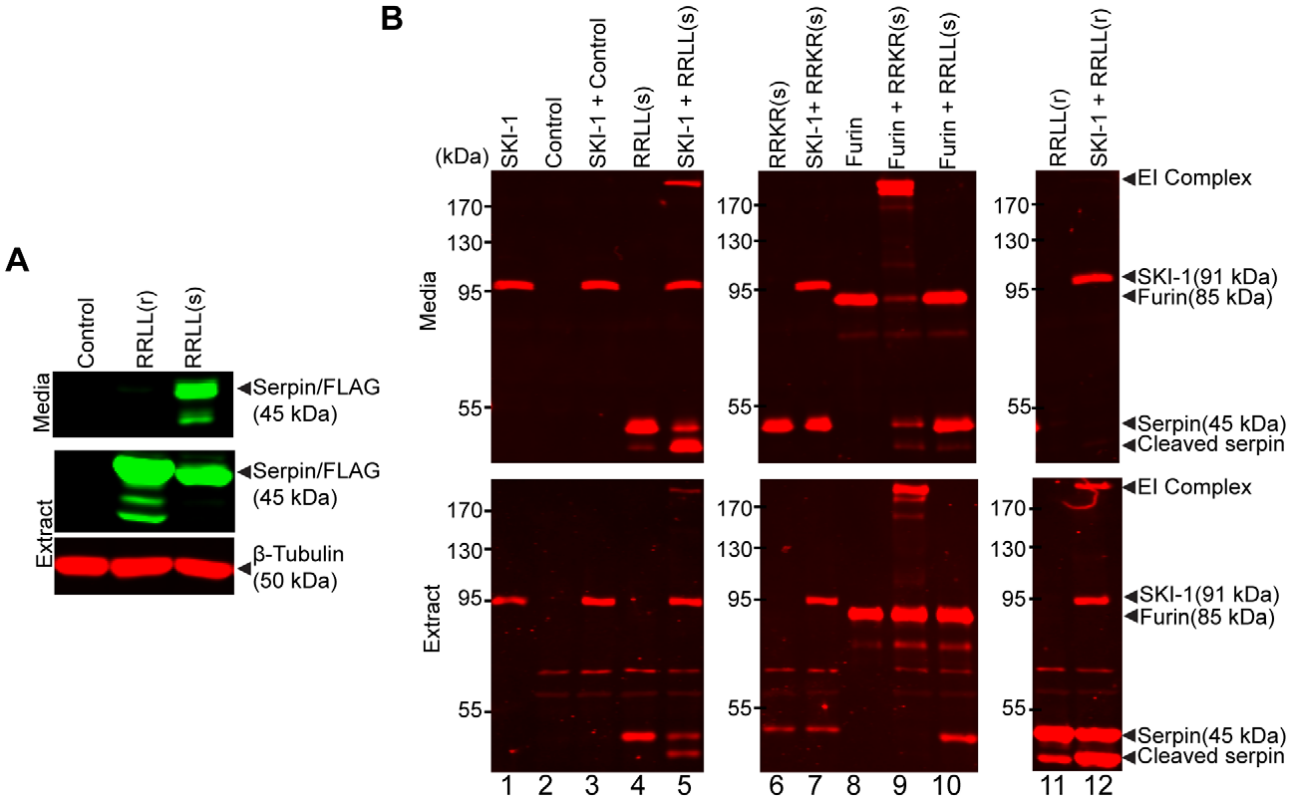
Cellular expression and serpin-like properties of recombinant adenovirus-expressed Spn4A variants. (A) The cellular expression of serpin variants Spn4A.RRLL(r) and (s) were examined using infectious adenoviral-mediated expression (moi 50) in Huh-7.5.1 cells. An empty adenovirus vector (Ad-Empty) was used as a control. After 48 hours, cell media (upper panel) and lysates (lower panels) were subjected to Western blot analysis. Spn4A variants were detected with mouse anti-FLAG antibody and, to ensure equal loading of samples, extracts were also probed with rabbit anti-β-tubulin antibody. (B) Huh-7.5.1 cells were infected with recombinant adenovirus expressing the His- and FLAG-tagged Spn4A variants indicated or the Ad-Empty control for 72 hours. Media alone (upper panels) or cell extracts (lower panels) lysed in RIPA buffer were combined with recombinant His-tagged SKI-1/S1P or His-tagged furin for 30 minutes at 30°C. Samples were prepared for Western blot analysis and probed with mouse anti-His antibody to detect SDS- and heat-stable protease-serpin complex formation as described in the Materials and Methods. Representative Western blots of at least 2 individual experiments are shown.

The serpin-secretion profile in Huh-7.5.1 cells was examined by Western blotting of cell lysates and extracellular media. As expected, a prominent 45-kDa band was detected in Ad-Spn4A.RRLL(r)-infected and Ad-Spn4A.RRLL(s)-infected cell lysates using anti-FLAG antibody (Figure 2A, lanes 2 and 3). Furthermore, as hypothesized, the 45-kDa band of Spn4A.RRLL(r) was found only in lysed cell extracts and was not found secreted into extracellular media (Figure 2A, lane 2). Conversely, the 45-kDa band of Spn4A.RRLL(s) was detected in both whole cell extracts and in cell media (Figure 2A, lane 3). These results confirmed the robust intracellular expression of intact FLAG-tagged recombinant serpins (45-kDa protein band) and the differential secretion of Spn4A.RRLL(r) and Spn4A.RRLL(s) in Huh-7.5.1 cells.

### Serpin-like properties and intrinsic specificity of Spn4A.RRLL(r) and Spn4A.RRLL(s)

A stable, acyl-enzyme complex is formed between a protease and a functional inhibitory serpin following RCL cleavage. This allows for detection of the high molecular weight, heat-stable, and SDS-stable enzyme-inhibitor (EI) complex by standard SDS-PAGE and Western blot [54], [55], [58], [62]. To determine if Spn4A.RRLL(s) is a functional and selective inhibitor of SKI-1/S1P, recombinant Spn4A variants (furin- and SKI-1/S1P-directed inhibitors) were expressed in Huh-7.5.1 cells for 72 hours. Cell media and extracts containing recombinant serpins were then harvested and incubated with purified recombinant His-tagged human SKI-1/S1P or furin (Figure 2B) as described previously [47], [54], [58]. Reaction mixtures were analyzed by Western blot and probed for EI complex formation with anti-His antibody (Figure 2B) and anti-FLAG antibody (Figure S3). The results shown in Figure 2B clearly demonstrate EI complex formation between recombinant SKI-1/S1P and Spn4A.RRLL(s) in cell media and in cell extracts (lane 5, upper and lower panels). As expected, Spn4A.RRLL(s) did not form a complex with furin (Figure 2B, lane 10, upper and lower panels), whereas the furin-directed serpin Spn4A.RRKR(s) formed an EI complex with furin but not with SKI-1/S1P in cell media and extracts (Figure 2B, lanes 9 and 7, respectively, upper and lower panels). Lysed cellular extracts expressing Spn4A.RRLL(r) also demonstrated EI complex formation with SKI-1/S1P (Figure 2B, lane 12, bottom panel). The results of our biochemical analysis confirmed the serpin-like properties of recombinant Spn4A.RRLL(r) and (s) biosynthesized in human hepatoma cells and the selectivity of Spn4A.RRLL(s) against SKI-1/S1P. Importantly, Spn4A.RRLL(s) inhibits SKI-1/S1P by a suicide substrate mechanism and forms a kinetically trapped heat- and SDS-stable complex with SKI-1/S1P as is characteristic of other physiological serpin-protease pairs [58], [63].

### Spn4A.RRLL(s) is a potent inhibitor of SKI-1/S1P-mediated endoproteolytic cleavage of SREBPs, of their downstream effector gene expression, and of intracellular cholesterol-ester accumulation

**Figure 3:**
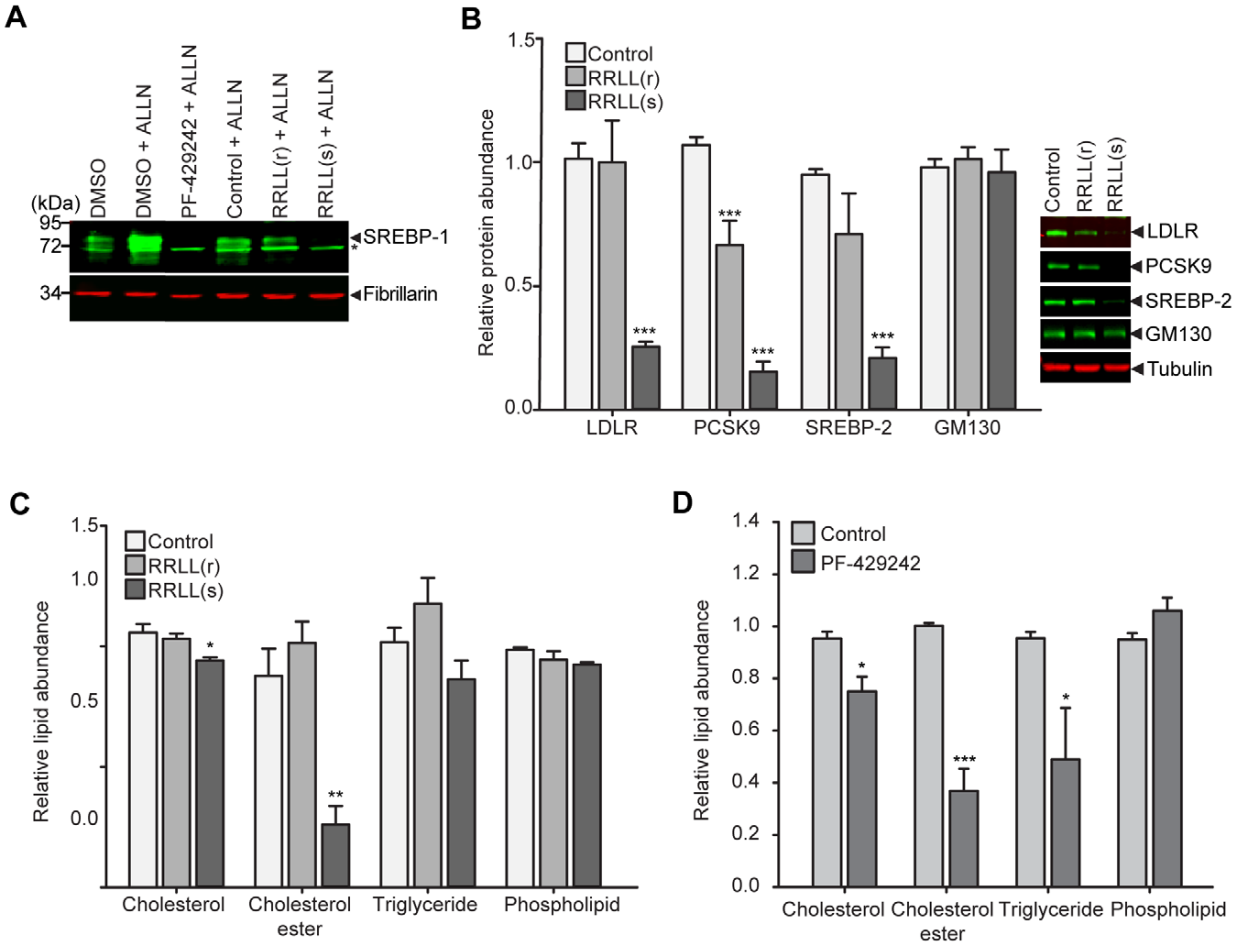
PF-429242 and Spn4A.RRLL(s) inhibit SREBP activation and reduce neutral lipid abundance in Huh-7.5.1 cells. (A) Huh-7.5.1 cells were treated with DMSO or 10 µM PF-429242 for 24 hours or infected with Ad-Empty (control), Ad-Spn4A.RRLL(r) or Ad-Spn4A.RRLL(s) for 48 hours. Cell extracts were harvested and subjected to nuclear fractionation as described in the Materials and Methods. Nuclear extracts were probed for N-terminal SREBP-1 expression and for enrichment of the nucleolar marker, fibrillarin. The * denotes a non-specific band detected by polyclonal anti-SREBP-1 antibody. (B) Huh-7.5.1 cells were grown in LPDS-supplemented media for 24 hours and were then infected with Ad-Spn4A.RRLL(r), Ad-Spn4A.RRLL(s), or Ad-Empty (control) for 72 hours; cell extracts were harvested and subjected to Western blot analysis. Anti-PCSK9, LDLR, SREBP-2, and GM130 antibodies were used to detect protein expression levels, and β-tubulin was probed for normalizing band intensities. Values are plotted relative to protein expression in control cells (left panel). The right panel shows representative Western blots for the effect of serpin on each protein examined. (C) Cells were treated with DMSO (control) or 10 µM PF-429242 for 24 hours. The compound was removed and the cells incubated for an additional 24 hours. Cells were then harvested and the relative levels of intracellular lipids were determined in treated cells relative to control cells. (D) Huh-7.5.1 cells were infected with Ad-Spn4A.RRLL(r), Ad-Spn4A.RRLL(s), or Ad-Empty (control) for 72 hours. Cells were then harvested and the relative levels of intracellular lipids were determined in treated cells relative to the control. In (A), a representative Western blot of 3 independent experiments is shown. For (B), (C), and (D), results (mean ± SEM) from 3 independent experiments are shown. *p<0.05; **p<0.01; ***p<0.005.

To confirm that expression of Spn4A.RRLL(s) in Huh-7.5.1 cells inhibits endogenous SKI-1/S1P-mediated cleavage of SREBP molecules, we examined nuclear SREBP-1 protein levels in cells infected with adenovirus-expressed Spn4A variants. As a positive control, we also treated cells with the selective, reversible, and competitive small-molecule inhibitor of SKI-1/S1P: PF-429242 [64], [65]. This compound was recently synthesized and characterized both *in vitro* and *in vivo* for its anti-lipidemic properties including efficient inhibition of nuclear SREBP accumulation. As previously described [64], [66], the calpain inhibitor, alpha-N-acetyl-Leu-Leu-Nle-CHO (ALLN), was employed to facilitate the detection and accumulation of the N-terminal fragment of SREBP-1 in the nucleus. An anti-fibrillarin antibody was used to positively identify the nuclear fractions (Figure 3A) [67]. As expected, Western blotting of nuclear extracts from cells treated with 10 µM of PF-429242 (PF-429242 + ALLN), using an antibody against the N-terminal fragment of SREBP-1, revealed a complete block of SREBP-1 accumulation in the nucleus (Figure 3A). Nuclear extracts from cells infected with Ad-Spn4A.RRLL(s) (RRLL(s) + ALLN) also exhibited a dramatic decrease in nuclear SREBP-1 accumulation when compared to Ad-Empty (control + ALLN) and Ad-Spn4A.RRLL(r) (RRLL(r) + ALLN)-infected cells. These results confirm that expression of recombinant Spn4A.RRLL(s) in the secretory pathway of Huh-7.5.1 cells inhibits SREBP-1 processing by SKI-1/S1P.

Next, to determine whether the Spn4A.RRLL(s)-mediated reduction in nuclear SREBPs was associated with a concomitant decrease in the protein levels in SREBP-target genes, we examined the fate of three SREBP-dependent gene products, SREBP-2, LDLR, and PCSK9. We investigated these host cell proteins because of their proposed contribution to HCV entry (LDLR and PCSK9) and propagation (SREBP-2) [19], [68], [69]. A time course analysis of cells expressing Spn4A.RRLL(s) in complete media showed the most significant block in SREBP-regulated LDLR expression after 72 hours (Figure S4). As HCV is known to induce SREBP activation [19], [31], [32], we then analyzed the expression of SREBP-regulated proteins under SREBP-activated conditions (Figure 3B). Huh-7.5.1 cells were depleted of exogenous sterols for 24 hours to induce SREBP transport from ER-to-Golgi prior to infection with Ad-Empty, Ad-Spn4A.RRLL(r), or Ad-Spn4A.RRLL(s). The levels of LDLR, PCSK9, and SREBP-2 (all regulated by nuclear SREBPs [70]-[73]) were then measured using Western blot analysis of lysed cell extracts (Figure 3B). After 72 hours of Spn4A.RRLL(s) expression, mature LDLR (160 kDa) levels were reduced by 74% compared to Ad-Empty-treated cells. Similarly, an 85% block in mature PCSK9 (60 kDa) expression and a 79% reduction in full-length SREBP-2 expression were observed. No significant reductions in LDLR or SREBP-2 levels following Spn4A.RRLL(r) treatment were observed. Interestingly, a significant reduction in PCSK9 expression was detected in Spn4A.RRLL(r)-expressing cells (Figure 3B). The expression of β-tubulin and of the Golgi marker GM130 were not affected by Spn4A.RRLL(r) or Spn4A.RRLL(s) expression (Figure 3B). These results confirm that expression of Spn4A.RRLL(s) in Huh-7.5.1 cells specifically inhibits the SREBP pathway including target genes identified as cellular cofactors affecting HCV infection.

A critical function of the SREBP pathway and the genes that it regulates is to control lipid homeostasis [36]. We investigated the impact of inhibiting SKI-1/S1P using both Spn4A.RRLL(s) and PF-429242 on total intracellular lipid levels, specifically cholesterol, cholesterol-esters, triglycerides, and phospholipids (Figure 3C and 3D). Among the cell lipids examined, Spn4A.RRLL(s) and PF-429242 had the most dramatic impact on cholesterol-ester levels, a major constituent of cellular LDs [5]; these were reduced by 74% in Spn4A.RRLL(s)-treated cells compared to control (Ad-Empty)-treated cells (Figure 3C). Similarly, PF-429242 reduced cholesterol-ester levels by ∼ 63% compared to control cells treated with DMSO (Figure 3D). A 14% reduction in triglycerides was also induced by Spn4A.RRLL(s), although this reduction did not reach significance, whereas PF-429242 caused a significant 51% reduction in total intracellular triglycerides. A significant 5% reduction in free cholesterol levels was also observed in Spn4A.RRLL(s)-treated cells and a 25% reduction was observed in PF-429242-treated cells compared to respective controls. No significant reductions in phospholipid levels were detected (Figure 3C and 3D). These results suggest that sustained inhibition of SKI-1/S1P-mediated cleavage and activation of SREBP causes increased cellular utilization of lipid stores.

### Expression of secretory pathway Spn4A.RRLL(s) in Huh-7.5.1 cells dramatically reduces the abundance of cellular lipid storage droplets

**Figure 4:**
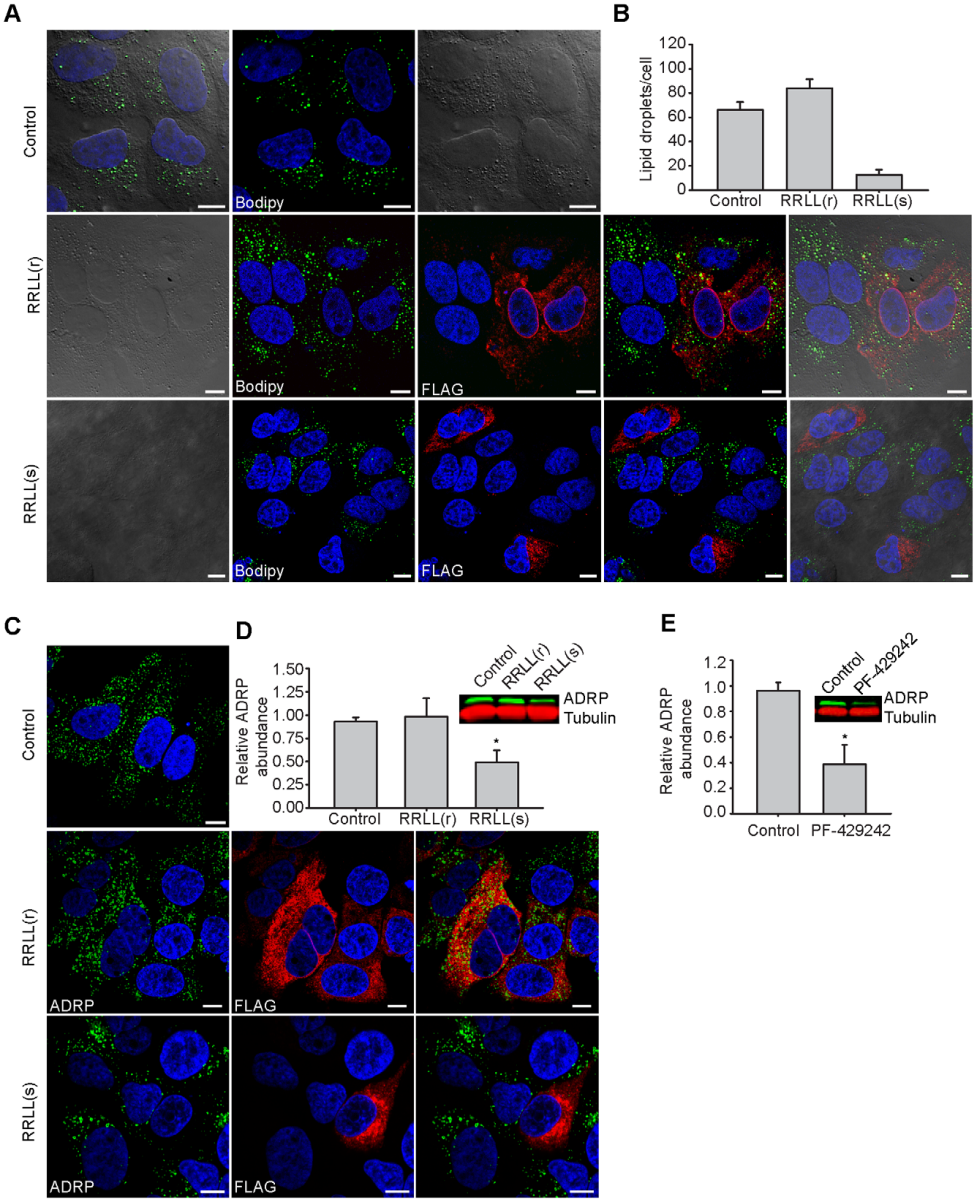
LD abundance is dramatically reduced in Huh-7.5.1 cells expressing Spn4A.RRLL(s). (A-C) Huh-7.5.1 cells were infected with Ad-Empty (control), Ad-Spn4A.RRLL(r), or Ad-Spn4A.RRLL(s) for 72 hours. Fixed cells were stained for cell nuclei using Hoechst dye (blue) and probed for expression of Spn4A variants using mouse anti-FLAG antibody (red). (A) LDs were detected using BODIPY 493/503 (green), and images were acquired using a Leica TCSSP5 confocal microscope. (B) MetaMorph imaging software was used to quantify the number of BODIPY-stained LDs in control cells (n = 23) and individual cells expressing Spn4A.RRLL(r) (n = 21) or Spn4A.RRLL(s) (n = 15). Acquisition and analysis were performed using the same intensity and threshold settings across all images. (C) The LD marker ADRP was detected in cells treated with Spn4A.RRLL(r), Spn4A.RRLL(s), and Ad-Empty (control) using rabbit anti-ADRP antibody (green), and images were obtained using an Olympus Fluoview FV1000 laser scanning confocal microscope. (D) Huh-7.5.1 cells infected with Ad-Empty (control), Ad-Spn4A.RRLL(r), or Ad-Spn4A.RRLL(s) for 72 hours were harvested and subjected to SDS-PAGE and Western blot analysis. Mouse anti-ADRP antibody was used to detect protein expression levels in serpin-treated cells compared to control-treated cells. Relative protein expression was quantified by normalizing to β-tubulin expression. The inset shows a representative Western blot. (E) Huh-7.5.1 cells were treated with DMSO (control) or with 10 µM PF-429242 for 24 hours, the compound was removed, and the cell lysates were harvested after an additional 48 hours. Relative ADRP expression (normalized to β-tubulin) in inhibitor-treated cells compared to control cells was quantified by subjecting total cell lysates to Western blot analysis. Values are plotted relative to protein expression in control cells, which are set to 1. Results (mean ± SEM) from 3 independent experiments are shown. *p<0.05.

LDs are dynamic intracellular lipid storage compartments made up of triglyceride and cholesterol esters, surrounded by a phospholipid membrane and associated with specific marker proteins including adipose differentiation-related protein (ADRP), also known as perlipin 2 [74]. Because SREBP activation controls the expression of genes directly involved in intracellular fatty acid and cholesterol biosynthesis (reviewed in [36]) and because cholesterol-ester levels were reduced by Spn4A.RRLL(s), we investigated the effect of serpin-mediated SKI-1/S1P inhibition on cellular LD abundance. Fluorescence microscopy was used to determine the relative abundance of LDs stained with BODIPY 493/503 in Huh-7.5.1 cells infected with Ad-Empty (control), Ad-Spn4A.RRLL(r), and Ad-Spn4A.RRLL(s). After 72 hours, the level of BODIPY-stained LDs in Spn4A.RRLL(s)-expressing cells was dramatically reduced compared to empty vector-treated cells (Figure 4A). By contrast, Spn4A.RRLL(r)-expressing cells had no apparent reduction in LD size or abundance compared to control-treated cells (Figure 4A). Quantification of confocal microscopy images demonstrated that, on average, LD abundance was reduced by 80% in Spn4A.RRLL(s)-expressing cells compared to controls (Figure 4B). The effect of Spn4A.RRLL(s) expression on cytosolic LD abundance was confirmed by visualizing the LD marker ADRP/perilipin 2 using confocal microscopy (Figure 4C) and by using quantitative Western blot (Figure 4D). Spn4A.RRLL(s) was observed to reduce ADRP/perilipin 2 protein expression by 50% compared with control cells (Figure 4D), whereas there was no reduction in ADRP/perlipin 2 expression following Spn4A.RRLL(r) treatment. We subsequently confirmed that reduced cellular LD levels were due to inhibition of SKI-1/S1P using 10 µM PF-429242 whereupon ADRP/perilipin 2 levels were reduced by 62% compared to control DMSO-treated cells (Figure 4E). These results confirm that Spn4A.RRLL(s)- and PF-429242-mediated inhibition of SKI-1/S1P enzymatic activity dramatically reduces intracellular LD abundance in Huh-7.5.1 cells.

### Expression of secretory pathway Spn4A.RRLL(s) in Huh-7.5.1 cells results in a dose-dependent inhibition of HCV infection

**Figure 5:**
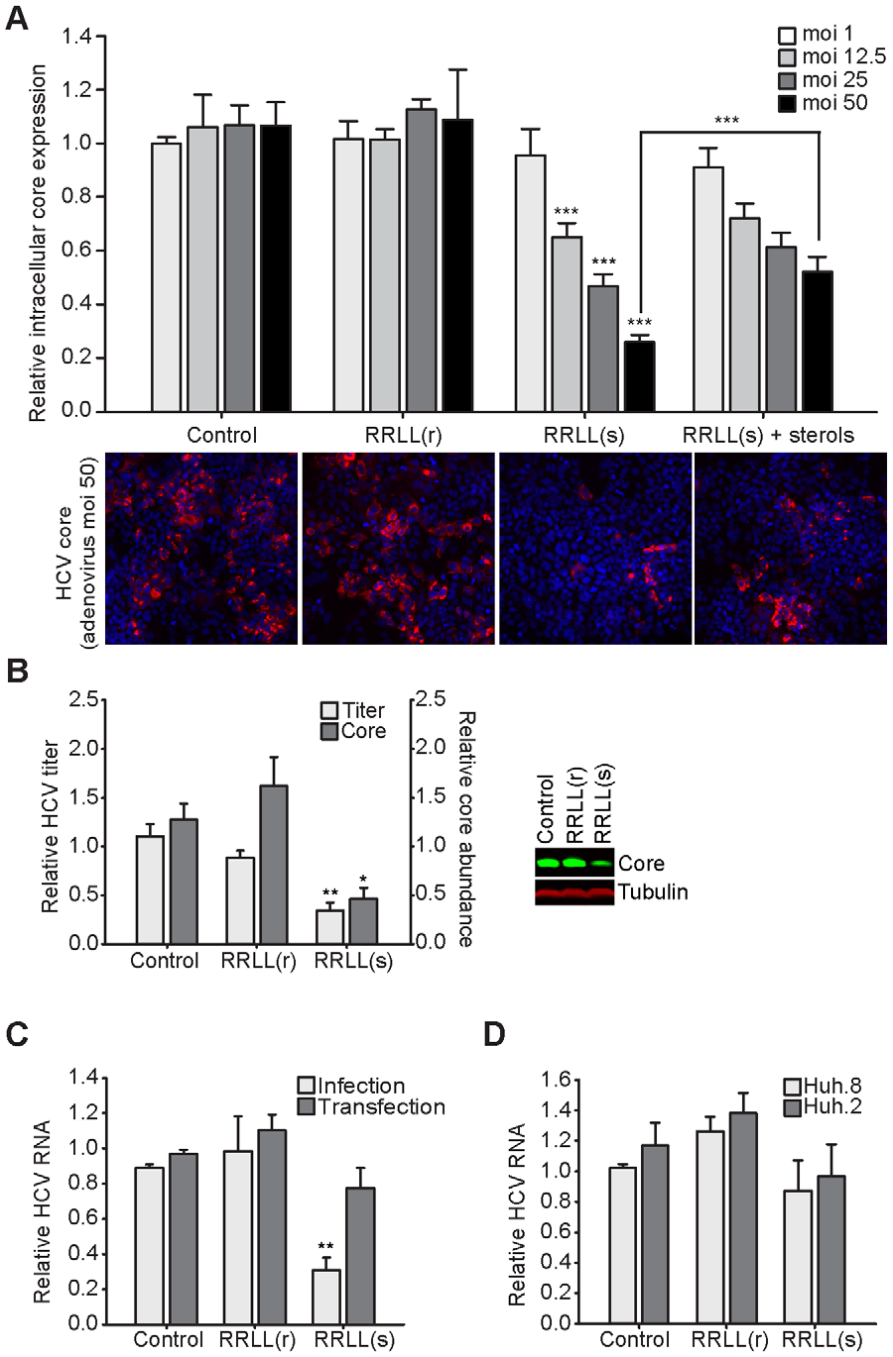
Inhibition of SKI-1/S1P using Spn4A.RRLL(s) results in a dose-dependent inhibition of HCV infection in Huh-7.5.1 cells. (A) Huh-7.5.1 cells were infected with moi (1 – 50) of Ad-Empty (control), Ad-Spn4A.RRLL(r), or Ad-Spn4A.RRLL(s) for 48 hours in regular media or media supplemented with sterols. Treated cells were infected with HCV (moi 0.1) and fixed 72 hours post-HCV-infection. Cells, probed with HCV anti-core antibody (red) and stained with Hoechst dye (nuclei; blue), were quantified using Cellomics HCS to determine the percentage of HCV-infected cells. Sample images of cells infected with adenovirus moi 50 acquired with Cellomics HCS using the 10 X objective are shown below the graph. (B) Huh-7.5.1 cells were infected with Ad-Empty (control), Ad-Spn4A.RRLL(r), or Ad-Spn4A.RRLL(s) (moi 50) for 48 hours in complete media and then infected with HCV (moi 0.1) for 72 hours. Relative HCV-core expression (normalized to β-tubulin) in serpin-treated cells compared to control-treated cells was quantified by examining total cell lysates using Western blot analysis. Infectious HCV titer in the extracellular media was also determined and relatively quantified. (C) Control and serpin-treated cells were infected with HCV or transfected with genomic HCV RNA, and total cellular RNA was harvested 72 hours later. HCV RNA levels, normalized to β-actin transcript levels, were relatively quantified in cell extracts using real-time PCR. (D) Huh.2 and Huh.8 replicon-harbouring cells were treated with serpin-expressing and control adenoviruses for 5 days before total RNA was harvested. HCV RNA levels, normalized to β-actin transcript levels, were relatively quantified in cell extracts using real-time PCR. All values are expressed as relative HCV levels in serpin-treated cells compared to control-treated cells. Results (mean ± SEM) from at least 3 independent experiments are shown. Statistical significance was calculated for Ad-Spn4A.RRLL(r) or Ad-Spn4A.RRLL(s) infection compared to Ad-Empty infection at the same moi. *p<0.05; **p<0.01; ***p<0.005.

Since the SREBP signaling pathway is induced by HCV-encoded proteins during infection [19], [30]-[32], we tested the effect of inhibiting this pathway on the HCV lifecycle in human hepatoma cells. Huh-7.5.1 cells were treated with increasing moi (1–50) of Ad-Spn4A.RRLL(r), Ad-Spn4A.RRLL(s), or Ad-Empty (control) for 48 hours in complete media with or without exogenously added sterols followed by 72 hours of infection with HCV. The number of HCV-infected cells, as evidenced by positive core protein expression, was measured using Cellomics HCS (Figure 5A). It was determined that Spn4A.RRLL(s) expression inhibited HCV infection in a dose-dependent manner compared to control-treated cells. HCV infection was not significantly reduced in cells infected with Ad-Spn4A.RRLL(s) at a moi of 1. A moi of 12.5, however, caused a 40% reduction, a moi of 25 caused a 60% reduction, and a moi of 50 caused a 75% reduction in the number of HCV-infected cells compared to controls. Spn4A.RRLL(r) had no significant impact on HCV infection up to adenovirus moi 50 when compared to the control (Figure 5A). Supplementing sterol and lipid metabolites significantly restored infectivity when cells were treated with moi 50 of Ad-Spn4A.RRLL(s), where a 2-fold increase in HCV infection compared to non-supplemented cells was observed (Figure 5A). These results show that the anti-HCV activity of Spn4A.RRLL(s) is, at least in part, associated with its capacity to decrease intracellular lipid stores within the host cells.

The anti-HCV properties of Spn4A.RRLL(s) were confirmed and extended by further virological studies on cells pre-treated with our serpin-based inhibitors for 48 hours prior to 72 hours of HCV infection (Figure 5B and 5C). First, quantitative Western blot analysis revealed a 64% reduction in the expression of intracellular HCV core protein in Spn4A.RRLL(s)-expressing cells compared to the control (Figure 5B). Similarly, Spn4A.RRLL(s) treatment was found to reduce extracellular infectious HCV titers by 76% (Figure 5B). A 65% reduction in intracellular HCV RNA levels was also observed using quantitative PCR (QPCR analysis (Figure 5C). Spn4A.RRLL(r) expression did not significantly impact any aspect of the HCV lifecycle examined (Figure 5A, 5B and 5C). These results demonstrate that inhibition of SKI-1/S1P-mediated proteolytic activation of SREBP molecules using secretory pathway protein-based inhibitors is an effective antiviral strategy to robustly block HCV infection in Huh-7.5.1 cells.

### Expression of secretory pathway Spn4A.RRLL(s) does not compromise HCV replication in human hepatoma cells

To determine if a decrease in intracellular HCV RNA in Spn4A.RRLL(s)-treated cells (Figure 5C) is due to reduced viral replication or alternatively due to reduced HCV entry, we examined cells transfected directly with total genomic HCV RNA for 3 days following 48 hours of adenovirus-mediated serpin expression. We found that under these experimental conditions, when receptor-mediated HCV entry was bypassed, no significant changes in intracellular HCV RNA levels were detected by QPCR (Figure 5C). Examination of total cell extracts by Western blot revealed that HCV core levels are not reduced following HCV RNA transfection in serpin-treated cells (Figure S5), confirming that Spn4A.RRLL(s) does not interfere with HCV replication when HCV entry is bypassed.

The impact of Spn4A.RRLL(s) treatment on HCV replication was further investigated using HCV subgenomic replicons [75]. Human hepatoma cells harbouring stable HCV replicons encoding wild-type NS5A (Huh.8 cells) or NS5A with an adaptive mutation (Huh.2 cells) were treated with recombinant adenoviruses for 5 days. Total RNA levels were then harvested and the level of HCV RNA was quantified using QPCR analysis. No significant differences were observed between HCV replicon levels treated with Spn4A.RRLL(s), Spn4A.RRLL(r), and the control (Ad-Empty) (Figure 5D). These results confirm that Spn4A.RRLL(s) does not inhibit HCV replication. These findings, in addition to the measured decrease in LDLR expression presented in Figure 3B, strongly suggest that the robust reduction in intracellular HCV RNA levels observed in Spn4A.RRLL(s) pre-treated cells prior to HCV infection is, at least in part, due to reduced viral attachment or entry.

### The anti-HCV properties of PF-429242

**Figure 6:**
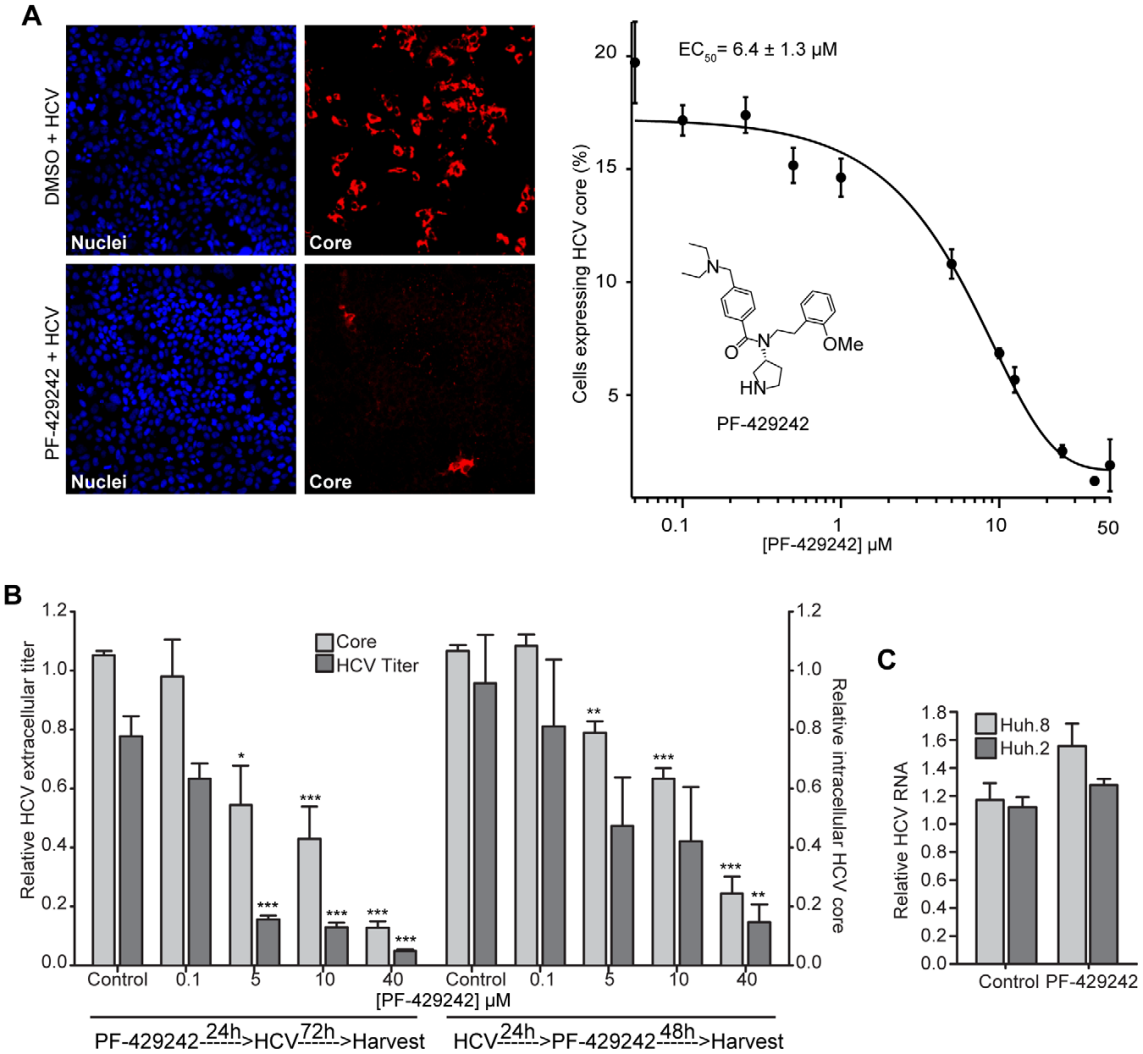
Inhibition of SKI-1/S1P using PF-429242 results in a dose-dependent inhibition of HCV infection, pre- and post-establishment of viral infection in Huh-7.5.1 cells. (A) Huh-7.5.1 cells were treated with various concentrations (0.01 to 50 µM) of PF-429242 for 24 hours. The inhibitor was removed and the cells were then infected with HCV (moi 0.1) in complete media for 48 hours. Cells, probed with HCV anti-core antibody (red) and stained with Hoechst dye (nuclei; blue), were quantified using Cellomics HCS to determine the percentage of HCV-infected cells. The average EC_50_ value from 3 independent experiments is displayed above the graph. Sample images of cells treated with DMSO or 50 µM PF-429242 acquired with Cellomics HCS using the 10 X objective are shown to the left of the graph. (B) Cells were treated with different concentrations (0.1 to 40 µM) of PF-429242 either 24 hours before (left half of graph) or 24 hours after (right half of graph) infection with HCV (moi 0.1). At 72 hours post-HCV infection, cells were fixed and probed with HCV anti-core antibody (red) and stained with Hoechst dye (nuclei; blue) to determine the percentage of HCV-infected cells (core) using Cellomics HCS. Also, media from treated and HCV-infected cells was harvested and the amount of extracellular, infectious HCV was titered (HCV titer; FFU/ml). Results (mean ± SEM) from 3 independent experiments are shown. (C) Huh.8 and Huh.2 replicon-harbouring cells were treated with DMSO (control) or 10 µM PF-429242 for 72 hours before total RNA levels were harvested. HCV RNA levels, normalized to β-actin transcript levels, were relatively quantified in cell extracts using real-time PCR. Results (mean ± SEM) from 2 independent experiments are shown. Statistical significance was calculated for PF-429242 treated cells compared to DMSO-treated cells. *p<0.05; **p<0.01; ***p<0.005.

Next, we wanted to test whether extracellularly applied PF-429242 would effectively inhibit the endoproteolytic activity of secretory pathway SKI-1/S1P and reduce HCV infection in Huh-7.5.1 cells. First, using an MTS-based cell viability assay, we confirmed that no major cytotoxic effects occur in Huh-7.5.1 cells treated with up to 50 µM of PF-429242 (Figure S6). Next, cells were treated with increasing concentrations (0.05 µM to 50 µM) of PF-429242 for 24 hours before the cell media was replaced and cells were infected for 48 hours with HCV. The number of HCV-infected cells, indicated by positive core protein expression, was measured using Cellomics HCS (Figure 6A, left panel). As expected, host cell pretreatment with PF-429242 resulted in a dose-dependent decrease in the number of HCV-infected cells (EC_50_ 6.4±1.3 µM) (Figure 6A, right panel). A near complete block in HCV infection was observed following treatment with 40 µM of inhibitor (Figure 6A, right panel).

To examine further the impact of PF-429242 prophylactic treatment on the different stages of the HCV lifecycle, we next tested the effect of 24-hour PF-429242 pre-treatment on the production of infectious progeny virions, which are normally detected 72 hours post-HCV infection in Huh-7.5.1 cells. Under these experimental conditions, a 16-fold reduction in extracellular viral titers was observed (Figure 6B, left panel) with an EC_50_ concentration of 1.03±0.3 µM. Interestingly, a single prophylactic pre-treatment of Huh-7.5.1 cells with 40 µM PF-429242 was sufficient to maintain a 10-fold decrease of intracellular HCV core production 72 hours post-infection (EC_50_ value of 6.5±4.8 µM) (Figure 6B, right panel). These results indicated that pre-treatment of host cells with PF-429242 was sufficient to robustly block HCV infection by preventing HCV entry and/or downstream production of infectious progeny virions.

To elucidate the lifecycle stage compromised by treatment with PF-429242, we tested the effect of the compound when added after the HCV entry stage in Huh-7.5.1 cells. We hypothesized that if only the entry stage of HCV lifecycle is blocked when SREBP cleavage is inhibited by PF-429242, addition of PF-429242 24 hours post-HCV infection should have no impact on the progression of HCV infection. To test this hypothesis, cells were first infected with HCV for 24 hours, allowing uninterrupted HCV entry and establishment of infection. HCV-containing media was then removed, and the cells were treated with 0.05 µM to 50 µM of PF-429242. In this scenario, HCV infection was reduced by up to 78% in cells treated with 40 µM PF-429242 compared to DMSO-treated cells (Figure 6B, right panel). Infectious virus production (extracellular viral titer) was reduced by ∼90% following treatment with 40 µM of inhibitor (EC_50_ of 6.0±3.5 µM) (Figure 6B, right panel). These results indicate that SKI-1/S1P inhibition also inhibits post-entry stages of the HCV lifecycle.

Because PF-429242 and Spn4A.RRLL(s) decrease the abundance of LD components, we hypothesized that HCV assembly, rather than HCV replication, can also blocked by SKI-1/S1P inhibition. To support this hypothesis, Huh.8 and Huh.2 cells were treated with 10 µM PF-429242 for 72 hours. No significant changes in HCV subgenomic RNA levels in either cell line (Figure 6C) treated with PF-429242 were observed (compared to DMSO-treated control). In summary, with the lack of effect of PF-429242 on HCV replicon levels and comparing the two sets of data presented in Figure 6B, which demonstrate a strong antiviral effect of PF-429242 when added either pre- or post-HCV inoculation, we can propose that pharmacological inhibition of SKI-1/S1P endoproteolytic activity by PF-429242 impacts late assembly stages of the HCV lifecycle.

## Discussion

**Figure 7:**
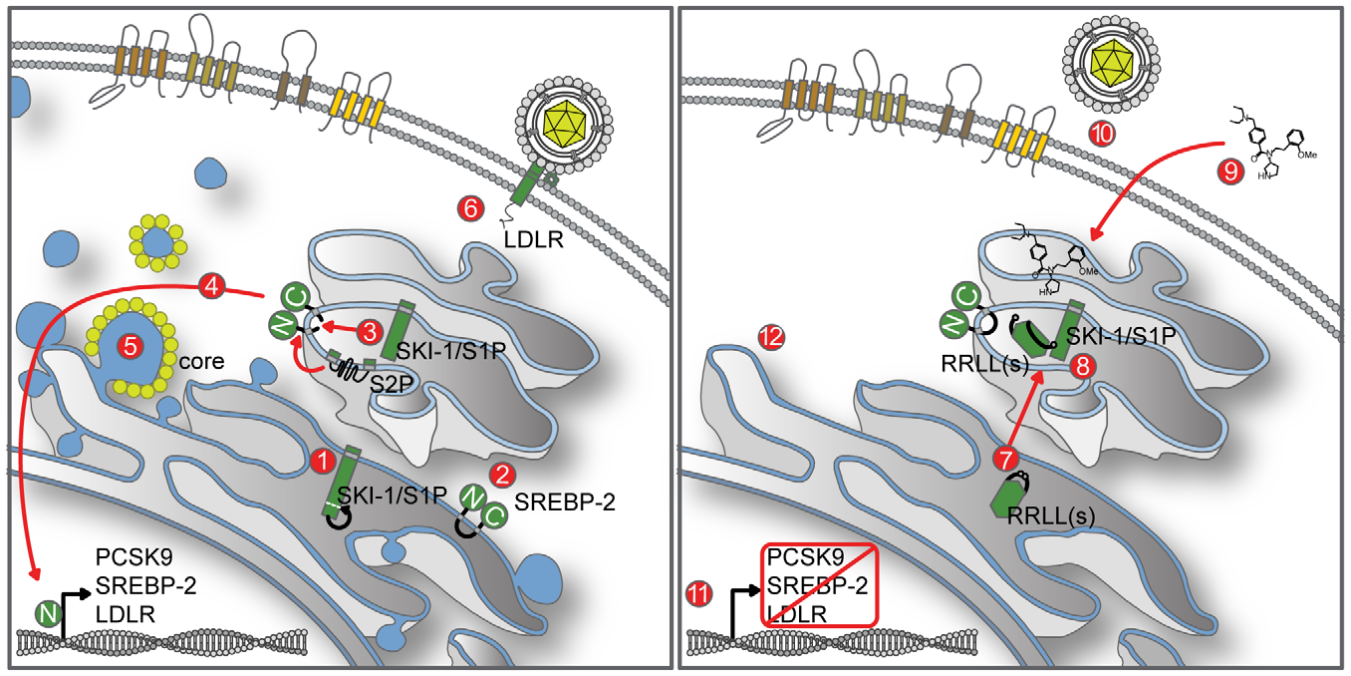
SKI-1/S1P is a novel potential target for indirect-acting antiviral agents against HCV infection. **(1)** The inactive SKI-1/S1P zymogen is biosynthesized in the ER and traffics to the Golgi apparatus following intramolecular autocatalytic maturation of the proenzyme [35], [46], [99], [100]. **(2)** During HCV infection, the SREBP pathway is activated by a variety of molecular mechanisms [19], [30]–[32]. **(3)** For SREBP to activate genes involved in lipid biosynthesis, its N-terminal domain must be released through sequential endoproteolytic cleavage first by SKI-1/S1P and then by S2P [37], [38]. **(4)** The released N-terminal domain translocates to the nucleus and activates various aspects of lipid metabolism [36]. **(5)** Activation of lipid biosynthesis increases LD formation where the HCV core protein localizes to orchestrate HCV assembly and subsequent secretion [8], [10], [16]. **(6)** Biosynthesis of LDLR, a proposed receptor for HCV entry, is also activated by SREBP signaling [69], [79], [101]. **(7)** Spn4A.RRLL(s) is a secretory pathway-expressed serpin (Figure 1). **(8)** Spn4A.RRLL(s) interacts and forms a covalent complex with enzymatically active SKI-1/S1P molecules (Figure 2 and S3) in the Golgi apparatus preventing SKI-1/S1P-mediated endoproteolytic cleavage of SREBP protein (Figure 3A). **(9)** A small-molecule inhibitor PF-429242 also efficiently inhibits SKI-1/S1P endoproteolytic activity (Figure 3A). **(10)** SKI-1/S1P inhibition blocks expression of the putative HCV receptor, LDLR (Figure 3B and S4), and reduces HCV entry (Figure 5). **(11)** The expression of other SREBP-regulated genes, such as PCSK9 and SREBP-2, are also blocked (Figure 3B). **(12)** Downstream lipid synthesis is interrupted resulting in overall reduced intracellular cholesterol-ester and triglyceride abundance (Figure 3C and 3D). **(12)** This is then detected as a decrease in LD abundance (Figure 4), which impedes assembly and secretion of infectious HCV particles.

It is now well established that hijacking of host-cell biosynthetic pathways by human enveloped viruses is a shared molecular event essential for the viral lifecycle [76]. The next frontier is identifying common, critical, host cell pathways that are hijacked by pathogenic human viruses, in order to develop broad-spectrum, host-directed antivirals with novel mechanisms of action. In this study, we hypothesized that targeting cellular enzymes acting as master regulators of lipid homeostasis could represent a powerful approach to developing a novel class of antiviral agents against infection associated with human enveloped viruses such as HCV, whose replication and pathogenesis depend on the interaction with lipid droplets (LDs) [8]. In the case of HCV, overstimulation of host lipid metabolism in the liver during viral infection promotes cholesterol intracellular storage in host LDs, a critical cellular event for the HCV lifecycle that leads to steatosis of the liver in HCV-infected patients [8], [18], [77]. One such master regulator of cholesterol metabolic pathways is the host proprotein convertase SKI-1/S1P [36], [78]. SKI-1/S1P plays a critical role in the proteolytic activation of SREBPs, which control expression of key enzymes of cholesterol and fatty-acid biosynthesis [41], [53]. Here, we report that strategic manipulation of cellular SKI-1/S1P activity levels by protein-based or small-molecule protease inhibitors provides a means of effectively inhibiting HCV infection (JFH-1 strain) of Huh-7.5.1 cells in a dose-dependent manner. Furthermore, we reveal the common molecular and cellular mechanisms of action of the SKI-1/S1P inhibitors and demonstrate that they act as negative modulators of cytoplasmic LD abundance (Figure 7), an organelle central to HCV assembly [8] and liver steatosis.

### SKI-1/S1P-directed inhibitors are a new class of lipid droplet-modulating agents

To investigate the biological consequences of inhibiting SKI-1/S1P endoprotease activity on biochemical pathways of lipid homeostasis hijacked by HCV, we first employed a protein-based inhibitor strategy. We engineered and developed a novel, recombinant adenovirus expressing an effective and specific secretory pathway, SKI-1/S1P-directed serpin, Spn4A.RRLL(s). We showed that Spn4A.RRLL(s) forms a kinetically trapped heat- and SDS-stable complex with SKI-1/S1P molecules characteristic of other physiological serpin-protease pairs. We then demonstrated that it blocks the SKI-1/S1P-mediated cleavage of endogenous SREBP-1 and the expression of SREBP downstream effector gene products (e.g., SREBP-2, LDLR, and PCSK9). The SREBP target gene products identified in Spn4A.RRLL(s)-treated cells are involved in lipid homeostasis and reported to participate in HCV-host interactions. SREBP-2, along with the other SREBP isoforms SREBP-1a and -1c, are activated by HCV-encoded proteins or during HCV infection [19], [31]. PCSK9 has been implicated in HCV infection through its regulation of two HCV entry factors: CD81 and LDLR [68]. Although the specific role of LDLR in HCV infection is unclear, increasing evidence indicates that LDLR promotes attachment and uptake of lipoprotein-associated HCV particles into hepatocytes [29], [69], [79], [80].

In addition to blocking SREBP-mediated up-regulation of hepatic genes during HCV infection, we hypothesized that blocking SKI-1/S1P endoprotease activity would also compromise cellular lipid storage. Analysis of intracellular lipid content and cytoplasmic LD abundance in Spn4A.RRLL(s)-expressing cells confirmed our hypothesis. In addition, we observed a decrease in ADRP/perilipin 2 abundance in Spn4A.RRLL(s)-expressing cells compared to control-treated cells. The physiological role of ADRP has yet to be fully established but it plays an important role in LD structure and formation. Interestingly, ADRP is degraded through the proteasome-dependent pathway during regression of lipid-storing cells, indicating that when ADRP is not bound to LDs, such as in SKI-1/S1P-inhibited cells, it will be susceptible to rapid proteasomal degradation [81]. Clinical studies have demonstrated that there is a correlation between the level of ADRP and the degree of hepatocyte steatogenesis in humans [82]. In addition, ADRP is found to be up-regulated in fatty liver in humans and in mice with liver steatosis [83]. Collectively, these observations suggest that inhibition of SKI-1/S1P offers an attractive therapeutic target for reducing HCV-induced liver steatosis.

### SKI-1/S1P is a potential target for indirect-acting antiviral agents against HCV infection

As hypothesized, inhibiting SKI-1/S1P-mediated SREBP endoproteolytic cleavage events using Spn4A.RRLL(s) resulted in a dose-dependent decrease in HCV infection. We demonstrated that HCV core expression and HCV RNA levels are reduced in Spn4A.RRLL(s)-expressing cells following HCV infection, leading to a robust reduction in extracellular infectious HCV particle release. Western blotting also confirmed that HCV core protein post-translational processing was unaltered by serpin expression because the size of the protein was unaltered. Supplementing Spn4A.RRLL(s)-expressing cells with compounds such as mevalonate, oleate, and cholesterol resulted in an incomplete rescue of HCV infection, suggesting that the antiviral activity of our protein-based inhibitor cannot be explained solely by the decreased availability of lipids in these cells. This also supports our hypothesis that reduced LDLR levels may compromise HCV entry into Spn4A.RRLL(s)-treated hepatocytes. LDLR, a well-established SREBP-regulated gene, has been repeatedly shown to support HCV entry into hepatocytes [69], [80]. Also, no significant reductions in HCV RNA levels were observed in Spn4A.RRLL(s)-treated cells harbouring subgenomic HCV replicons or following full-length genomic HCV RNA transfection in Huh-7.5.1 cells. Altogether, these studies indicate that the observed decline in HCV RNA levels following HCV infection may result from compromised HCV entry.

To gain further insight into the different stages of the viral lifecycle targeted by our SKI-1/S1P inhibitor, we used an active-site-directed small-molecule inhibitor of SKI-1/S1P, PF-429242. This pharmacologic inhibitor of SKI-1/S1P has recently been characterized for its effectiveness in blocking cleavage of SREBP-2, for blocking expression of SREBP-activated genes, and also for inhibiting arenavirus glycoprotein processing [64], [84]. In contrast to our recombinant adenovirus-expressed serpins, PF-429242 can be added extracellularly to rapidly inhibit SKI-1/S1P. This allows us to study the biological impact of blocking SKI-1/S1P-dependent pathways during both early and late stages of HCV infection.

We first confirmed a reduction in abundance of neutral lipids and ADRP/perilipin 2 expression in PF-429242-treated cells. Then, we confirmed that inhibition of SKI-1/S1P using PF-429242 blocks HCV infection and extracellular infectious virus production in a dose-dependent manner. The anti-HCV activity of PF-429242 is very robust and particularly striking. A single 24-hour pre-treatment with the compound was sufficient to block HCV infection, and the antiviral effect of PF-429242 was still apparent 72 hours post-treatment. Importantly, pharmacological treatment of already infected HCV cells resulted in a 90% reduction of HCV virus production. Similar to Spn4A.RRLL(s) treatment, PF-429242 did not reduce HCV RNA levels in the two stable subgenomic replicon cell lines that were examined. This, in combination with the observed reduction in abundance of a central organelle (LD) involved in HCV assembly, and the reduction in HCV particle secretion in cells treated 24 hours after HCV inoculation, supports PF-429242 as an inhibitor of late stages of the HCV lifecycle, i.e., during assembly or egress. Taken all together, these results indicate that inhibiting SKI-1/S1P can interrupt the HCV lifecycle at multiple stages of viral infection both preventing naïve cells from becoming infected and preventing virus release from already infected cell populations. Thus, developing more effective active-site-directed SKI-1/S1P small-molecule inhibitors (< nM range) with better pharmacokinetic properties [64] could lead to novel indirect-acting antiviral treatment options for HCV-infected patients [42], [76], [85], [86]. Importantly, inhibiting the SREBP pathway in HCV-infected cells, which have exacerbated lipid production and which are steatotic, may relieve symptoms caused by chronic HCV infection in addition to blocking viral infection [87].

In conclusion, pharmacologic inhibition of SKI-1/S1P offers a very promising avenue for the development of novel anti-HCV therapeutics (Figure 7). On one hand, targeting a host cell master molecular switch such as SKI-1/S1P with a novel class of drugs compromising multiple stages of the virus lifecycle would have the main advantage of making it more difficult for the virus to develop escape mutations [86], [88]. On the other hand, the toxicity issues associated with the inhibition of host cell proteases such as SKI-1/S1P [89] could be addressed by using adjunctive therapy, combining our novel class of lipid-modulating agents with the current standard of care or with the appropriate synergistic direct-acting antivirals [85], [86].

Finally, our results reveal that targeting host LD biogenesis by inhibiting SKI-1/S1P endoproteolytic activity may have far-reaching applications in the therapeutic treatment of other important human *Flaviviridae* viruses such as dengue virus, whose replication and pathogenesis also depend on the interaction with lipid droplets [7].

## Materials and Methods

### Cell culture and reagents

Human hepatoma Huh-7.5.1 cells were kindly provided by Dr. Francis Chisari (Scripps Research Institute, La Jolla, CA, USA) [61]. Huh.8 (Con1/SG-Neo) cells, supporting HCV genotype 1b subgenomic replicon, and Huh.2 (Con1/SG-Neo: S2197P) cells, supporting replicon with NS5A adaptive mutation, were provided by Dr. Charles Rice (The Rockefeller University, New York, NY, and Apath, LLC, St. Louis, MO, USA) [75]. Cultured cells were maintained in Dulbecco's Modified Eagle Medium (DMEM) supplemented with 1% penicillin, streptomycin, glutamine, non-essential amino acids, 4-(2-hydroxyethyl)-1-piperazineethanesulfonic acid (HEPES), and 10% fetal bovine serum (FBS) (Gibco/Invitrogen, Burlington, ON, Canada) or 10% lipoprotein-depleted serum (LPDS) (Biomedical Technologies Inc., Stoughton, MA, USA). Huh.8 and Huh.2 were also maintained under selection with 750 µM Geneticin (Invitrogen). Bovine serum albumin (BSA), saponin, cholesterol, α-mevalonic acid, lactone, and oleaic acid were obtained from Sigma-Aldrich Corp. (St. Louis, MO, USA). Formaldehyde, 37% w/v was obtained from Fischer Scientific (Pittsburgh, PA, USA).

### Antibodies and dyes

A mouse anti-core antibody (1∶500, Abcam, Cambridge, MA, USA) was employed for detecting HCV infection. Antibodies used to detect cellular proteins included rabbit anti-human proprotein convertase subtilisin/kexin type 9 (PCSK9) (1∶500, Abcam), rabbit anti-LDLR (1∶250, Fitzgerald Industries International, Inc., Concord, MA, USA), mouse anti-SREBP-2 (1∶200, Santa Cruz Biotechnology, Santa Cruz, CA, USA), rabbit anti-adipose differentiation-related protein (ADRP) (1∶50, Abcam), mouse anti-ADRP (1∶20, Progen, Heidelberg, Germany), rabbit anti-β-tubulin (1∶3000, Abcam), mouse anti-β-tubulin (1∶500, Lab Vision Corporation, Montréal, QC, Canada), mouse anti-GM130 (1∶200, BD Biosciences, Mississauga, ON, Canada), rabbit anti-SREBP-1, clone H160 (1∶200, Santa Cruz Biotechnology), and mouse anti-fibrillarin (1∶100, Abcam). Tagged proteins were detected using mouse anti-FLAG M2 (1∶500 for Western blot and 1∶50 for immunofluorescence, Sigma-Aldrich Corp.), rabbit anti-FLAG (1∶500 for Western blot and 1∶50 for immunofluorescence, Thermo Scientific, Nepean, ON, Canada), and mouse anti-His antibodies (1∶500, Applied Biological Materials, Richmond, BC, Canada). Secondary antibodies used for immunofluorescence were Alexa Fluor-488-conjugated or Alexa Fluor-568-conjugated donkey anti-mouse or donkey anti-rabbit antibodies (1∶100, Molecular Probes/Invitrogen). Secondary antibodies used for Western blot were IRDye 680-conjugated (red bands) or 800-conjugated (green bands) donkey anti-mouse or goat anti-rabbit antibodies (1∶10000, LI-COR Biosciences, Lincoln, NE, USA). Hoechst 33258 (10 µg/ml) and BODIPY (4,4-difluoro-1,3,5,7,8-pentamethyl-4-bora-3a,4a-diaza-s-indacene) 493/503 (1 µg/ml, Molecular Probes/Invitrogen) were used for detection of nuclei and LDs [90], respectively.

### HCV RNA and infectious stock production

A plasmid containing the cDNA of an HCV consensus clone isolated from a Japanese patient with fulminant hepatitis (JFH-1) (GenBank accession number AB047639) [91] cloned behind a T7 promoter (pJFH-1; a generous gift from Dr. Takaji Wakita, National Institute of Infectious Diseases, Tokyo, Japan) was used to generate genomic HCV RNA and infectious HCV stocks as previously described in [61].

### Transfection of HCV RNA

Purified HCV RNA was used to transfect Huh-7.5.1 cells as a means of studying HCV infection independently of receptor-mediated entry. Five micrograms of purified RNA was incubated with 10 µl of lipofectamine 2000 (Invitrogen) in minimal essential media (MEM) for 30 minutes. The RNA-lipid complexes were added to cells in MEM for 16 hours; then cells were washed with phosphate-buffered saline (PBS) and complete media was added for the remainder of the experiment.

### HCV titer determination

The amount of infectious HCV particles generated for viral stocks or in the described experiments was determined using a modified, previously described protocol [61]. Briefly, 1×10^4^ Huh-7.5.1 cells were plated in each well of a 96-well plate and infected with 10-fold serial dilutions of HCV-infected cell media. At 72 hours post-infection, cells were fixed and probed as described in the ArrayScan Quantification methods section. An ArrayScan VTI High Content Screening (HCS) Reader (Thermo Scientific) was used to acquire images of the entire group of infected wells. Titers were determined by manually counting foci (fluorescence forming units (FFU)) in the lowest dilutions with positive signal.

### Recombinant adenoviruses expressing His-/FLAG-tagged serpin variants

Spn4A (GenBank Accession number NM_165496; [92]) is the most potent and effective natural serpin of PCs identified to date [58]. For this reason, we selected Spn4A as a prototype of a protein inhibitor scaffold for engineering a secretory pathway SKI-1/S1P-directed inhibitor. First, the N-terminus of Spn4A was modified to encode the alpha-1-antitrypsin signal peptide (residues 1–24, pre-α_1_-AT) followed by a 6-His tag (HHHHHH) and FLAG-tag (DYKDDDDK) sequence (HF) [58]. The Spn4A-variants directed at furin contain the P4 – P1 cleavage sequence [49] Arg^P4^-Arg-Lys-Arg^P1↓^ (RRKR) in the reactive center loop (RCL). The ER “retained” (r) variant of the furin-directed serpin, designated Spn4A.RRKR(r) (Figure 1A), presents the His-Asp-Glu-Leu (HDEL) ER retention motif at the C-terminus. A “secreted” (s) variant of the serpin, Spn4A.RRKR(s), was generated by mutating the His codon in the HDEL sequence to a stop codon. SKI-1/S1P was reported to cleave the Lassa virus glycoprotein sequence Arg^P4^-Arg-Leu-Leu^P1↓^ (RRLL) [50]. Thus, to specifically target this enzyme, we altered the furin-directed RCL sequence to RRLL through site-directed mutagenesis using QuikChange (Stratagene, La Jolla, CA, USA) to generate Spn4A.RRLL(r) and Spn4A.RRLL(s) (Figure 1A and 1B). To produce Ad-Spn4A.RRKR(s), Ad-Spn4A.RRLL(r), and Ad-Spn4A.RRLL(s), all Spn4A constructs were cloned into adenovirus vectors (pShuttle) [55] and then were amplified by ViraQuest Inc. (North Liberty, IA, USA). Adenovirus titers were determined in human embryonic kidney (HEK293T) cells using an Adeno-X Rapid Titer kit (Clontech, Mountain View, CA, USA) [55]. Empty adenovirus (Ad-Empty), which was kindly provided by Dr. Jan Breslow (The Rockefeller University, New York, NY, USA) [93], was amplified by Viraquest Inc.

### *In silico* homology model of Spn4A.RRLL variant

The *Drosophila melanogaster* Spn4B sequence (GeneBank Accession number gi|24586105|ref|NP_524955.2) exhibits 34% sequence homology with the human neuroserpin (hNS), for which a crystal structure is available [94] in the Protein Data Bank (PDB ID: 3F5N). Of the five chains in this pentameric structure of hNS, chain B is most well resolved with the fewest missing residues, and it was used as the template for the homology model presented in Figure 1B. The model was built and refined using the SwissPDB Viewer. The C-alpha residues in this model structure align to 1.9 Å RMSD with reference to the hNS structure.

### Recombinant PCs and enzymatic assays

The first 997 amino acids of human SKI-1/S1P lacking the C-terminal transmembrane domain but containing a C-terminal 8-His-tag (PGDDDDKHHHHHHHHSGS) were expressed in Sf9 insect cells as previously described [47]. Two liters of cell culture supernatant were used for purification. Two hundred milliliters of 200 mM Tris/HCl pH 8.0, 500 mM NaCl was added, and then the pH was adjusted to pH 8.0 by further addition of 2 M NaOH. The resulting precipitate was removed by centrifugation at 10000 x g for 30 minutes and subsequently filtered through a glass filter. The cleared supernatant was then applied to a small (0.9 ml column volume) IMAC column (Ni-Sepharose, GE Healthcare, Freiburg, Germany) by continuous flow (1.0 ml/minutes). The column had previously been equilibrated in 50 mM Tris/HCl pH 8.0, 500 mM NaCl (buffer A), and bound recombinant SKI-1/S1P was eluted with a continuous gradient over 30 column volumes to buffer A plus 300 mM imidazole. Collected fractions were assayed for SKI-1/S1P enzymatic activity as previously described [47] using the paranitroanilide (p-NA) acetylated (Ac) tetrapeptidyl substrate Ac-RRLL-pNA [custom synthesized by Peptides International (Louisville, Kentucky, USA)]. The most active fractions were pooled. Concentration and buffer exchange to buffer A was then done using spin concentrators (Millipore, Billerica, MA, USA) with a molecular weight cutoff of 30 kDa. The final preparation was, after addition of 30% v/v glycerol, stored at -80°C and had a specific activity of 0.018 U/mg (measured as above). Recombinant His-tagged furin (0.432 mg/ml) was purchased from R & D Systems (Minneapolis, MN, USA), and reactions with adenovirus recombinant serpin were performed under the buffer conditions for furin assays as previously described [54], [58].

### Western blot analysis

Cultured cells were washed with ice-cold PBS and re-suspended in cold radioimmunoprecipitation assay (RIPA) buffer (50 mM Tris-HCl pH 8, 150 mM NaCl, 1% octylphenyl-polyethylene glycol [IGEPAL], 0.5% sodium deoxycholate, 0.1% sodium dodecyl sulfate [SDS] containing 1 X Complete, EDTA-free, protease inhibitor cocktail [Roche, Laval, QC, Canada]). Whole cell extracts were vortexed and then clarified by centrifugation at 12000 x g for 15 minutes. Soluble extracts mixed with 2 X sample buffer (62.5 mM Tris-HCl, pH 6.8, 25% glycerol, 2% SDS, 0.01% bromophenol blue, and 5% beta-mercaptoethanol) were electrophoresed on 8–15% SDS polyacrylamide gels and transferred to nitrocellulose membranes. Membranes were blocked in Odyssey blocking buffer (LI-COR Biosciences) for one hour, and proteins of interest were detected by probing with the appropriate primary and secondary antibodies diluted in Odyssey blocking buffer containing 0.1% Tween 20. Protein bands were detected and quantified using the Odyssey Infrared Imaging System (LI-COR Biosciences). All immunoblots were scanned at a wavelength of 700 nm for detecting IRDye 680 labeled antibodies and at a wavelength of 800 nm for IRDye 800CW conjugated antibodies [95], [96]. Signal intensities were quantified by means of the Odyssey software version 3.0. Beta-tubulin was always used as a loading control and for normalizing protein expression. Media samples analyzed for secreted Spn4A variants were taken directly from cultured cells, mixed with 2 X sample loading buffer, and subjected to the described Western blot analysis.

### Stable covalent acyl-enzyme complex (EI) formation between serpin variants and PCs

Huh-7.5.1 cells were plated at 5×10^5^ cells per well in a 6-well plate and infected after 24 hours with Ad-Spn4A.RRLL(s), Ad-Spn4A.RRLL(r), Ad-Spn4A.RRKR(s), or Ad-Empty at moi 50. After 72 hours, media and cell extracts (harvested in hypotonic buffer containing 20 mM Tris, pH 7.4, 10 mM MgCl_2,_ and 10 mM CaCl_2_) were mixed 1∶1 with enzyme reaction buffer (SK1-1/S1P buffer contains 25 mM Tris-HCl, 25 mM MES, pH 7.4, 2.5 mM CaCl_2_ [47]; furin buffer contains 100 mM HEPES, pH 7.5, 1 mM CaCl_2_, 0.5% Triton X-100 [54], [58], 1 X Complete EDTA-free protease inhibitor cocktail), and 11.6 ng/µl SKI-1/S1P or 2.4 ng/µl furin. The enzyme mixture was incubated at 30°C for 30 minutes, and the reaction was stopped with 12.5 mM EDTA (final concentration). After completion, products were resolved on a 10% SDS-gel. The high-molecular weight band (EI) was visualized as described above for Western blotting.

### ArrayScan quantification

In black flat-bottom 96-well plates (BD Biosciences), cells were plated (>10,000 cells/well) and infected as described in the methods below. Following infection, cells were fixed in 4% formaldehyde v/v diluted in PBS and blocked in PBS containing 3% BSA, 0.3% Triton X-100, and 10% FBS. Cells were first probed with HCV anti-core antibody (1∶500) in PBS containing 3% BSA and 0.3% Triton X-100 (Binding Buffer), then incubated with Alexa Fluor-568-conjugated donkey anti-mouse secondary antibody (1∶1000) and 10 µg/ml Hoechst dye. Cells were analyzed by a quantitative, high-throughput, fluorescence microscope system called the Cellomics ArrayScan VTI High Content Screening (HCS) Reader (Thermo Scientific) using the software Target Activation BioApplication (TABA). TABA was used to count the total number of cells (Hoechst-stained nuclei) and the percentage of those cells that were expressing HCV core (positive signal at 568 nm wavelength).

### Nuclear fractionation

On Day 1, 2.5×10^6^ Huh-7.5.1 cells were plated into 100 mm plates. On Day 2, cells were infected with recombinant adenoviruses expressing Spn4A.RRLL(r) or Spn4A.RRLL(s), or with Ad- Empty (moi 50). On Day 3, separate cells were treated with the inhibitor PF-429242 or the control DMSO. On Day 5, all samples (except the untreated control) were treated for 1 hour with 25 µM α-N-acetyl-Leu-Leu-Nle-CHO ((2S)-2-[[(2S)-2-acetamido-4-methylpentanoyl]amino]-4-methyl-N-[(2S)-1-oxohexan-2-yl]pentanamide) (Sigma-Aldrich Corp.). Nuclear fractionation of cell lysates was performed at 4°C as described previously with modifications [64], [66]. Cells were harvested in 400 µl buffer C (10 mM HEPES/KOH, pH 7.6, 10 mM KCl, 1.5 mM MgCl_2_, 1 mM EDTA, 1 mM EGTA, 250 mM sucrose) containing 1 X complete protease inhibitor cocktail (Roche). To shear DNA, the cells were passed 20 times through a 23-gauge needle. The lysate was centrifuged at 1100 x g for 7 minutes and the resulting supernatant was centrifuged again at 25000 x g for 60 minutes to obtain the membrane pellet. The pellet containing membrane-bound SREBP-1 was re-suspended in 75 µl of SDS-lysis buffer (10 mM Tris HCl, 100 mM NaCl, 1% SDS, 1 mM EDTA, 1 mM EGTA, pH 6.8). The pellet from the 1100 x g spin was re-suspended in 100 µl buffer D (20 mM HEPES/KOH, 420 mM NaCl, 1.5 mM MgCl_2_, 2.5% glycerol, 1 mM EDTA, 1 mM EGTA, pH 7.6) containing 1 X complete protease inhibitor cocktail. Nuclear pellets were rocked for 1 hour, after which the samples were centrifuged at 25000 x g for 60 minutes to obtain the clarified supernatant containing the nuclear fraction.

### Confocal microscopy and immunofluorescence

After Huh-7.5.1 cells were seeded onto coverslips for 24 hours, they were infected with adenovirus (moi 50) for 72 hours. Cells were fixed in 4% v/v formaldehyde in PBS, then permeabilized and blocked in PBS containing 0.05% saponin (wash buffer) and 1% BSA (binding buffer). Blocking of cells stained with BODIPY 493/503 was done in the presence of 0.2 M glycine to reduce background fluorescence. Cells were probed with primary antibodies in binding buffer, then incubated with a secondary antibody, Hoechst dye (10.0 µg/ml), and BODIPY 493/503 (1.0 µg/ml; when indicated) diluted in PBS. Cells were mounted onto slides with an anti-fade solution and sealed with clear nail polish. The slides were then imaged using a Leica TCS SP5 confocal microscope (Leica Microsystems, Wetzlar, Germany) or an Olympus Fluoview FV1000 laser scanning confocal microscope (Olympus Corporation, Tokyo, Japan) [95]–[97]. Leica MM AF Software (Leica Microsystems) was used to count the number of LDs (green channel) in cells expressing Spn4A.RRLL(r) (n = 21) or Spn4A.RRLL(s) (n = 15) (cells positive in the red channel). LDs in cells treated with Ad-Empty (n = 23) were also enumerated. All quantified images were acquired using the same laser intensity and gain settings, and LDs were enumerated by applying the same threshold setting to each image.

### Adenovirus infections

Huh-7.5.1 cells were infected with recombinant adenovirus at different moi in complete media with or without exogenous sterols (50 µM sodium mevalonate, 20 µM sodium oleate, 5.0 µg/ml cholesterol). After 48 hours, the cells were infected with HCV moi 0.1 or transfected with purified HCV genomic RNA for 72 hours, and then cells were analyzed by Cellomics ArrayScan HCS, or total RNA was isolated from cell extracts using the RNeasy plus kit (Qiagen, Mississauga, ON, Canada) including on-column DNase digestion. Media from treated and infected cells were harvested for HCV titer determination as described above. To examine the Spn4A.RRLL(s) mediated block in PCSK9, LDLR, and SREBP-2 expression, Huh-7.5.1 cells were grown in media supplemented with LPDS for 24 hours, infected with adenovirus variants, and harvested 72 hours later.

### Quantitative real-time (Q) PCR

Purified total RNA was reverse transcribed to cDNA using TaqMan reverse transcription reagents (random hexamers; Applied Biosystems, Foster City, CA, USA). Real-time quantitative PCR was carried out using Brilliant II Fast QPCR reagents (Stratagene, La Jolla, CA, USA) according to the manufacturer's instructions on an Mx3005P QPCR system (Stratagene). Online ProbeFinder software (Roche Applied Science) was used to find primers that would allow amplification of the HCV RNA 5′ end in combination with the Human Universal Probe Library from Roche (Roche Applied Science). For amplification of the HCV RNA 5′ region, 400 nM of both forward primer (5′-CATGGCGTTAGTATGAGTGTCG-3′) and reverse primer (5′-GGTTCCGCAGACCACTAT-3′) were used in combination with 200 nM of probe #75 from the Human Probe Library (Roche). HCV RNA levels were relatively quantified across samples and normalized to beta-actin RNA levels using 500 nM primers (forward: 5′- GCC CTG AGG CAC TCT TCC and reverse: 5′ GGA TGT CCA CGT CAC ACT TC-3′) and 250 nM probe (5′AC TCC ATG CCC AGG AAG GAA GGC-3′ with a 5′ Cy5 fluorophore and 3′ black hole quencher).

### Cytotoxicity assay

Cell viability was determined using CellTiter 96 AQueous One Solution Cell Proliferation Assay (Promega, Madison, WI, USA). This assay employs a tetrazolium compound [3-(4,5-dimethylthiazol-2-yl)-5-(3-carboxymethoxyphenyl)-2-(4-sulfophenyl)-2H-tetrazolium, inner salt; MTS], which is bio-reduced by cells into a colored formazan product that can be detected in tissue culture media at 490 nm wavelength [98].

### Treatment with PF-429242

PF-429242, an active site-directed small-molecule inhibitor of SKI-1/S1P [64], [84], was synthesized by Dr. Peter Chua at the Center for Drug Research and Development (CDRD) at the University of British Columbia (Vancouver, BC, Canada) according to previously described protocols [65]. To investigate the antiviral activity of the small molecule, Huh-7.5.1 cells were treated with PF-429242 for 24 hours. After 24 hours of treatment, the media was removed and then cells were infected with HCV (moi 0.1) for 48 or 72 hours. Alternatively, cells were first infected with HCV for 24 hours; then, the media was removed and replaced with media containing various concentrations of PF-429242 for a further 48 hours. Intracellular HCV infection levels were determined using Cellomics HCS ArrayScan. Infectious extracellular titers were determined in media of 72-hour HCV-infected cells.

### Intracellular lipid quantification

To measure the level of intracellular lipids following 72-hour recombinant adenovirus expression or 48-hour PF-429242 treatment, cellular extracts were harvested in 1% triton-X 100 in PBS (for phospholipid, cholesterol, and protein assay) or 5% triton-X 100 in H_2_O (for triglyceride assay). To extract triglycerides, samples were slowly heated to 90°C and brought to room temperature, twice. Total cholesterol, cholesterol esters (Amplex Red Cholesterol Assay kit, Invitrogen), phospholipids, and triglycerides (EnzChrom, BioAssay Systems, Hayward, CA, USA) were quantified using commercially available kits. Lipid levels were normalized to cellular protein content (DC Protein Assay, Bio-Rad, Hercules, CA, USA).

### Curvefitting, half-maximal effective concentration (EC_50_) determination and statistics

The sigmoidal fit function in Igor Pro software (WaveMetrics, Inc., Portland, OR, USA) was used for fitting HCV and PF-429242 inhibition curves and for determining EC_50_ values. The reported EC_50_ values are the average of the values calculated from three independent experiments plus or minus the standard deviation. The student's *t*-test (unpaired) was used to calculate significance, which is represented in the figures by the following notation: * denotes p<0.05, ** denotes p<0.01, and *** denotes p<0.005.

## Supporting Information

Figure S1 **Optimization of adenovirus-expressed Spn4A.RRLL(r) expression in Huh-7.5.1 cells.** Huh-7.5.1 cells were infected with moi 1, 12.5, 25, and 50 of the intracellularly retained serpin Ad-Spn4A.RRLL(r). Treated cells were fixed 48 hours post-infection and probed for serpin expression using mouse anti-FLAG antibody. Cell nuclei were stained with Hoechst dye to determine the total cell number. The percentage of Spn4A.RRLL(r)-expressing cells was quantified using Cellomics HCS. Results (mean ± SEM) from 2 independent experiments are shown.
(TIF)

Figure S2 **Effect of Spn4A variant treatment and HCV infection on Huh-7.5.1 cell growth.** Huh-7.5.1 cells were infected with various moi (1 – 50) of Ad-Empty, Ad-Spn4A.RRLL(r), or Ad-Spn4A.RRLL(s) for 48 hours in complete media. Treated cells were infected with HCV (moi 0.1) and fixed 72 hours post-infection. Fixed cells were probed with Hoechst dye to stain for cell nuclei, which were then quantified using Cellomics HCS to determine the relative number of cells in each well under the varying conditions. All values are expressed as relative cell number in serpin-treated cells compared to cells infected with Ad-Empty, which is set to 1. Results (mean ± SEM) from 3 independent experiments are shown.
(TIF)

Figure S3 **Serpin-like properties of recombinant adenovirus-expressed Spn4A variants expressed in Huh-7.5.1 cells.** Huh-7.5.1 cells were infected with recombinant adenovirus expressing the His- and FLAG-tagged Spn4A variants indicated or the Ad-Empty control for 72 hours. Media alone (upper panels) or cell extracts (lower panels) lysed in RIPA buffer were combined with recombinant His-tagged SKI-1/S1P [47] or His-tagged furin for 30 minutes at 30°C. Samples were prepared for Western blot analysis and probed with rabbit anti-FLAG antibody to detect SDS- and heat-stable protease-serpin complex formation and also to distinguish serpin bands from protease bands on the Western blots. All Western blots shown are representative of at least 2 independent experiments.
(TIF)

Figure S4 **Time course analysis of LDLR expression in Spn4A.RRLL(s)-treated cells.** Huh-7.5.1 cells were grown in complete media for 24 hours and then infected with Ad-Empty (control) or Ad-Spn4A.RRLL(s). Cell extracts were harvested for Western blot 24, 48, and 72 hours post-infection, and lysates were then subjected to Western blot. Anti-LDLR antibody was used to detect protein-expression levels in control- and Ad-Spn4A.RRLL(s)-treated cells, and β-tubulin was probed for normalizing LDLR expression. Values are plotted relative to LDLR expression in control (Ad-Empty)-treated cells, which was set to 1. The representative results of 2 independent experiments are shown.
(TIF)

Figure S5 **Spn4A.RRLL(s) does not block HCV core production post-transfection of HCV RNA.** Huh-7.5.1 cells were infected with Ad-Empty (control), Ad-Spn4A.RRLL(r), or Ad-Spn4A.RRLL(s) (moi 50) for 48 hours in complete media and then transfected with genomic HCV RNA for 72 hours. Relative HCV-core expression (normalized to β-tubulin) in serpin-treated cells compared to control-treated cells was quantified by examining total cell lysates using Western blot analysis. Results (mean ± SEM) from 2 independent experiments are shown. A representative Western blot is shown to the right of the graph.
(TIF)

Figure S6 **The effect of PF-429242 on cell viability.** Huh-7.5.1 cells were treated with DMSO (control) or various concentrations of PF-429242 for 24 hours before the inhibitor was removed, and fresh complete media was added to the cells for an additional 48 hours. The relative cytotoxicity of the compound was then determined using an MTS-based cell viability assay. The absorbance measured at 490 nm is proportional to the number of living cultured cells. Results (mean ± SEM) from 3 independent experiments are shown. Statistical significance was calculated for PF-429242-treated cells compared to DMSO-treated cells.
(TIF)

## References

[1] Manes S, del Real G, Lacalle RA, Lucas P, Gomez-Mouton C (2000). Membrane raft microdomains mediate lateral assemblies required for HIV-1 infection.. EMBO Rep.

[2] Sakamoto H, Okamoto K, Aoki M, Kato H, Katsume A (2005). Host sphingolipid biosynthesis as a target for hepatitis C virus therapy.. Nat Chem Biol.

[3] Nguyen DH, Hildreth JE (2000). Evidence for budding of human immunodeficiency virus type 1 selectively from glycolipid-enriched membrane lipid rafts.. J Virol.

[4] Shi ST, Lee KJ, Aizaki H, Hwang SB, Lai MM (2003). Hepatitis C virus RNA replication occurs on a detergent-resistant membrane that cofractionates with caveolin-2.. J Virol.

[5] Olofsson SO, Bostrom P, Andersson L, Rutberg M, Perman J (2009). Lipid droplets as dynamic organelles connecting storage and efflux of lipids.. Biochim Biophys Acta.

[6] Cheung W, Gill M, Esposito A, Kaminski CF, Courousse N (2010). Rotaviruses associate with cellular lipid droplet components to replicate in viroplasms, and compounds disrupting or blocking lipid droplets inhibit viroplasm formation and viral replication.. J Virol.

[7] Samsa MM, Mondotte JA, Iglesias NG, Assuncao-Miranda I, Barbosa-Lima G (2009). Dengue virus capsid protein usurps lipid droplets for viral particle formation.. PLoS Pathog.

[8] Miyanari Y, Atsuzawa K, Usuda N, Watashi K, Hishiki T (2007). The lipid droplet is an important organelle for hepatitis C virus production.. Nat Cell Biol.

[9] Boulant S, Targett-Adams P, McLauchlan J (2007). Disrupting the association of hepatitis C virus core protein with lipid droplets correlates with a loss in production of infectious virus.. J Gen Virol.

[10] Shavinskaya A, Boulant S, Penin F, McLauchlan J, Bartenschlager R (2007). The lipid droplet binding domain of hepatitis C virus core protein is a major determinant for efficient virus assembly.. J Biol Chem.

[11] Herker E, Harris C, Hernandez C, Carpentier A, Kaehlcke K (2010). Efficient hepatitis C virus particle formation requires diacylglycerol acyltransferase-1.. Nat Med.

[12] Shepard CW, Finelli L, Alter MJ (2005). Global epidemiology of hepatitis C virus infection.. Lancet Infect Dis.

[13] Grebely J, Matthews GV, Dore GJ (2011). Treatment of acute HCV infection.. Nat Rev Gastroenterol Hepatol.

[14] Robertson B, Myers G, Howard C, Brettin T, Bukh J (1998). Classification, nomenclature, and database development for hepatitis C virus (HCV) and related viruses: proposals for standardization. International Committee on Virus Taxonomy.. Arch Virol.

[15] Moradpour D, Penin F, Rice CM (2007). Replication of hepatitis C virus.. Nat Rev Microbiol.

[16] Boulant S, Douglas MW, Moody L, Budkowska A, Targett-Adams P (2008). Hepatitis C virus core protein induces lipid droplet redistribution in a microtubule- and dynein-dependent manner.. Traffic.

[17] Moradpour D, Englert C, Wakita T, Wands JR (1996). Characterization of cell lines allowing tightly regulated expression of hepatitis C virus core protein.. Virology.

[18] Bartenschlager R, Penin F, Lohmann V, Andre P (2011). Assembly of infectious hepatitis C virus particles.. Trends Microbiol.

[19] Waris G, Felmlee DJ, Negro F, Siddiqui A (2007). Hepatitis C virus induces proteolytic cleavage of sterol regulatory element binding proteins and stimulates their phosphorylation via oxidative stress.. J Virol.

[20] Kim K, Kim KH, Ha E, Park JY, Sakamoto N (2009). Hepatitis C virus NS5A protein increases hepatic lipid accumulation via induction of activation and expression of PPARgamma.. FEBS Lett.

[21] Perlemuter G, Sabile A, Letteron P, Vona G, Topilco A (2002). Hepatitis C virus core protein inhibits microsomal triglyceride transfer protein activity and very low density lipoprotein secretion: a model of viral-related steatosis.. FASEB J.

[22] Moriya K, Yotsuyanagi H, Shintani Y, Fujie H, Ishibashi K (1997). Hepatitis C virus core protein induces hepatic steatosis in transgenic mice.. J Gen Virol.

[23] Bach N, Thung SN, Schaffner F (1992). The histological features of chronic hepatitis C and autoimmune chronic hepatitis: a comparative analysis.. Hepatology.

[24] Adinolfi LE, Gambardella M, Andreana A, Tripodi MF, Utili R (2001). Steatosis accelerates the progression of liver damage of chronic hepatitis C patients and correlates with specific HCV genotype and visceral obesity.. Hepatology.

[25] Antunez I, Aponte N, Fernandez-Carbia A, Rodriguez-Perez F, Toro DH (2004). Steatosis as a predictive factor for treatment response in patients with chronic hepatitis C. P R Health Sci J.

[26] Soresi M, Tripi S, Franco V, Giannitrapani L, Alessandri A (2006). Impact of liver steatosis on the antiviral response in the hepatitis C virus-associated chronic hepatitis.. Liver Int.

[27] Negro F, Sanyal AJ (2009). Hepatitis C virus, steatosis and lipid abnormalities: clinical and pathogenic data.. Liver Int.

[28] Alvisi G, Madan V, Bartenschlager R (2011). Hepatitis C virus and host cell lipids: an intimate connection.. RNA Biol.

[29] Syed GH, Amako Y, Siddiqui A (2010). Hepatitis C virus hijacks host lipid metabolism.. Trends Endocrinol Metab.

[30] Park CY, Jun HJ, Wakita T, Cheong JH, Hwang SB (2009). Hepatitis C virus nonstructural 4B protein modulates sterol regulatory element-binding protein signaling via the AKT pathway.. J Biol Chem.

[31] Oem JK, Jackel-Cram C, Li YP, Zhou Y, Zhong J (2008). Activation of sterol regulatory element-binding protein 1c and fatty acid synthase transcription by hepatitis C virus non-structural protein 2.. J Gen Virol.

[32] Kim KH, Hong SP, Kim K, Park MJ, Kim KJ (2007). HCV core protein induces hepatic lipid accumulation by activating SREBP1 and PPARgamma.. Biochem Biophys Res Commun.

[33] Brown MS, Goldstein JL (2009). Cholesterol feedback: from Schoenheimer's bottle to Scap's MELADL..

[34] Yang T, Espenshade PJ, Wright ME, Yabe D, Gong Y (2002). Crucial step in cholesterol homeostasis: sterols promote binding of SCAP to INSIG-1, a membrane protein that facilitates retention of SREBPs in ER.. Cell.

[35] Nohturfft A, DeBose-Boyd RA, Scheek S, Goldstein JL, Brown MS (1999). Sterols regulate cycling of SREBP cleavage-activating protein (SCAP) between endoplasmic reticulum and Golgi.. Proc Natl Acad Sci U S A.

[36] Brown MS, Goldstein JL (1999). A proteolytic pathway that controls the cholesterol content of membranes, cells, and blood.. Proc Natl Acad Sci U S A.

[37] Sakai J, Rawson RB, Espenshade PJ, Cheng D, Seegmiller AC (1998). Molecular identification of the sterol-regulated luminal protease that cleaves SREBPs and controls lipid composition of animal cells.. Mol Cell.

[38] Rawson RB, Zelenski NG, Nijhawan D, Ye J, Sakai J (1997). Complementation cloning of S2P, a gene encoding a putative metalloprotease required for intramembrane cleavage of SREBPs.. Mol Cell.

[39] Hua X, Yokoyama C, Wu J, Briggs MR, Brown MS (1993). SREBP-2, a second basic-helix-loop-helix-leucine zipper protein that stimulates transcription by binding to a sterol regulatory element.. Proc Natl Acad Sci U S A.

[40] Briggs MR, Yokoyama C, Wang X, Brown MS, Goldstein JL (1993). Nuclear protein that binds sterol regulatory element of low density lipoprotein receptor promoter. I. Identification of the protein and delineation of its target nucleotide sequence.. J Biol Chem.

[41] Sato R (2010). Sterol metabolism and SREBP activation.. Arch Biochem Biophys.

[42] Nakamuta M, Yada R, Fujino T, Yada M, Higuchi N (2009). Changes in the expression of cholesterol metabolism-associated genes in HCV-infected liver: a novel target for therapy?. Int J Mol Med.

[43] Chang ML, Yeh CT, Chen JC, Huang CC, Lin SM (2008). Altered expression patterns of lipid metabolism genes in an animal model of HCV core-related, nonobese, modest hepatic steatosis.. BMC Genomics.

[44] Molloy SS, Anderson ED, Jean F, Thomas G (1999). Bi-cycling the furin pathway: from TGN localization to pathogen activation and embryogenesis.. Trends Cell Biol.

[45] Thomas G (2002). Furin at the cutting edge: from protein traffic to embryogenesis and disease.. Nat Rev Mol Cell Biol.

[46] Seidah NG (2011). The proprotein convertases, 20 years later.. Methods Mol Biol.

[47] Bodvard K, Mohlin J, Knecht W (2007). Recombinant expression, purification, and kinetic and inhibitor characterisation of human site-1-protease.. Protein Expr Purif.

[48] Pasquato A, Burri DJ, Traba EG, Hanna-El-Daher L, Seidah NG (2011). Arenavirus envelope glycoproteins mimic autoprocessing sites of the cellular proprotein convertase subtilisin kexin isozyme-1/site-1 protease.. Virology.

[49] Schechter I, Berger A (1967). On the size of the active site in proteases. I. Papain.. Biochem Biophys Res Commun.

[50] Lenz O, ter Meulen J, Klenk HD, Seidah NG, Garten W (2001). The Lassa virus glycoprotein precursor GP-C is proteolytically processed by subtilase SKI-1/S1P.. Proc Natl Acad Sci U S A.

[51] Beyer WR, Popplau D, Garten W, von Laer D, Lenz O (2003). Endoproteolytic processing of the lymphocytic choriomeningitis virus glycoprotein by the subtilase SKI-1/S1P.. J Virol.

[52] Bergeron E, Vincent MJ, Nichol ST (2007). Crimean-Congo hemorrhagic fever virus glycoprotein processing by the endoprotease SKI-1/S1P is critical for virus infectivity.. J Virol.

[53] Eberle D, Hegarty B, Bossard P, Ferre P, Foufelle F (2004). SREBP transcription factors: master regulators of lipid homeostasis.. Biochimie.

[54] Jean F, Stella K, Thomas L, Liu G, Xiang Y (1998). alpha1-Antitrypsin Portland, a bioengineered serpin highly selective for furin: application as an antipathogenic agent.. Proc Natl Acad Sci U S A.

[55] Jean F, Thomas L, Molloy SS, Liu G, Jarvis MA (2000). A protein-based therapeutic for human cytomegalovirus infection.. Proc Natl Acad Sci U S A.

[56] Whisstock JC, Silverman GA, Bird PI, Bottomley SP, Kaiserman D (2010). Serpins flex their muscle: II. Structural insights into target peptidase recognition, polymerization, and transport functions.. J Biol Chem.

[57] Huntington JA, Read RJ, Carrell RW (2000). Structure of a serpin-protease complex shows inhibition by deformation.. Nature.

[58] Richer MJ, Keays CA, Waterhouse J, Minhas J, Hashimoto C (2004). The Spn4 gene of Drosophila encodes a potent furin-directed secretory pathway serpin.. Proc Natl Acad Sci U S A.

[59] Pelham HR (1990). The retention signal for soluble proteins of the endoplasmic reticulum.. Trends Biochem Sci.

[60] Arnberg N (2009). Adenovirus receptors: implications for tropism, treatment and targeting.. Rev Med Virol.

[61] Zhong J, Gastaminza P, Cheng G, Kapadia S, Kato T (2005). Robust hepatitis C virus infection in vitro.. Proc Natl Acad Sci U S A.

[62] Richer MJ, Juliano L, Hashimoto C, Jean F (2004). Serpin mechanism of hepatitis C virus nonstructural 3 (NS3) protease inhibition: induced fit as a mechanism for narrow specificity.. J Biol Chem.

[63] Silverman GA, Whisstock JC, Bottomley SP, Huntington JA, Kaiserman D (2010). Serpins flex their muscle: I. Putting the clamps on proteolysis in diverse biological systems.. J Biol Chem.

[64] Hawkins JL, Robbins MD, Warren LC, Xia D, Petras SF (2008). Pharmacologic inhibition of site 1 protease activity inhibits sterol regulatory element-binding protein processing and reduces lipogenic enzyme gene expression and lipid synthesis in cultured cells and experimental animals.. J Pharmacol Exp Ther.

[65] Hay BA, Abrams B, Zumbrunn AY, Valentine JJ, Warren LC (2007). Aminopyrrolidineamide inhibitors of site-1 protease.. Bioorg Med Chem Lett.

[66] DeBose-Boyd RA, Brown MS, Li WP, Nohturfft A, Goldstein JL (1999). Transport-dependent proteolysis of SREBP: relocation of site-1 protease from Golgi to ER obviates the need for SREBP transport to Golgi.. Cell.

[67] Wu WW, Pante N (2009). The directionality of the nuclear transport of the influenza A genome is driven by selective exposure of nuclear localization sequences on nucleoprotein.. Virol J.

[68] Labonte P, Begley S, Guevin C, Asselin MC, Nassoury N (2009). PCSK9 impedes hepatitis C virus infection in vitro and modulates liver CD81 expression.. Hepatology.

[69] Owen DM, Huang H, Ye J, Gale M (2009). Apolipoprotein E on hepatitis C virion facilitates infection through interaction with low-density lipoprotein receptor. Virology..

[70] Goldstein JL, Brown MS (2009). The LDL receptor.. Arterioscler Thromb Vasc Biol.

[71] Jeong HJ, Lee HS, Kim KS, Kim YK, Yoon D (2008). Sterol-dependent regulation of proprotein convertase subtilisin/kexin type 9 expression by sterol-regulatory element binding protein-2.. J Lipid Res.

[72] Sato R, Inoue J, Kawabe Y, Kodama T, Takano T (1996). Sterol-dependent transcriptional regulation of sterol regulatory element-binding protein-2.. J Biol Chem.

[73] Sakai J, Duncan EA, Rawson RB, Hua X, Brown MS (1996). Sterol-regulated release of SREBP-2 from cell membranes requires two sequential cleavages, one within a transmembrane segment.. Cell.

[74] Brasaemle DL, Barber T, Wolins NE, Serrero G, Blanchette-Mackie EJ (1997). Adipose differentiation-related protein is an ubiquitously expressed lipid storage droplet-associated protein.. J Lipid Res.

[75] Blight KJ, McKeating JA, Rice CM (2002). Highly permissive cell lines for subgenomic and genomic hepatitis C virus RNA replication.. J Virol.

[76] Prussia A, Thepchatri P, Snyder JP, Plemper RK (2011). Systematic Approaches towards the Development of Host-Directed Antiviral Therapeutics.. Int J Mol Sci.

[77] Jones DM, McLauchlan J (2010). Hepatitis C virus: assembly and release of virus particles.. J Biol Chem.

[78] Brown MS, Goldstein JL (1997). The SREBP pathway: regulation of cholesterol metabolism by proteolysis of a membrane-bound transcription factor.. Cell.

[79] Agnello V, Abel G, Elfahal M, Knight GB, Zhang QX (1999). Hepatitis C virus and other flaviviridae viruses enter cells via low density lipoprotein receptor.. Proc Natl Acad Sci U S A.

[80] Mazumdar B, Banerjee A, Meyer K, Ray R (2011). Hepatitis C virus E1 envelope glycoprotein interacts with apolipoproteins in facilitating entry into hepatocytes.. Hepatology.

[81] Xu G, Sztalryd C, Lu X, Tansey JT, Gan J (2005). Post-translational regulation of adipose differentiation-related protein by the ubiquitin/proteasome pathway.. J Biol Chem.

[82] Straub BK, Stoeffel P, Heid H, Zimbelmann R, Schirmacher P (2008). Differential pattern of lipid droplet-associated proteins and de novo perilipin expression in hepatocyte steatogenesis.. Hepatology.

[83] Motomura W, Inoue M, Ohtake T, Takahashi N, Nagamine M (2006). Up-regulation of ADRP in fatty liver in human and liver steatosis in mice fed with high fat diet.. Biochem Biophys Res Commun.

[84] Urata S, Yun N, Pasquato A, Paessler S, Kunz S (2011). Antiviral activity of a small-molecule inhibitor of arenavirus glycoprotein processing by the cellular site 1 protease.. J Virol.

[85] Georgel P, Schuster C, Zeisel MB, Stoll-Keller F, Berg T (2010). Virus-host interactions in hepatitis C virus infection: implications for molecular pathogenesis and antiviral strategies.. Trends Mol Med.

[86] Gelman MA, Glenn JS (2010). Mixing the right hepatitis C inhibitor cocktail..

[87] Whisstock JC, Bottomley SP (2006). Molecular gymnastics: serpin structure, folding and misfolding.. Curr Opin Struct Biol.

[88] Tan SL, Pause A, Shi Y, Sonenberg N (2002). Hepatitis C therapeutics: current status and emerging strategies.. Nat Rev Drug Discov.

[89] Ye J (2011). Cell biology. Protease sets site-1 on lysosomes.. Science.

[90] Hinson ER, Cresswell P (2009). The antiviral protein, viperin, localizes to lipid droplets via its N-terminal amphipathic alpha-helix.. Proc Natl Acad Sci U S A.

[91] Kato T, Furusaka A, Miyamoto M, Date T, Yasui K (2001). Sequence analysis of hepatitis C virus isolated from a fulminant hepatitis patient.. J Med Virol.

[92] Han J, Zhang H, Min G, Kemler D, Hashimoto C (2000). A novel Drosophila serpin that inhibits serine proteases.. FEBS Lett.

[93] Maxwell KN, Fisher EA, Breslow JL (2005). Overexpression of PCSK9 accelerates the degradation of the LDLR in a post-endoplasmic reticulum compartment.. Proc Natl Acad Sci U S A.

[94] Takehara S, Onda M, Zhang J, Nishiyama M, Yang X (2009). The 2.1-A crystal structure of native neuroserpin reveals unique structural elements that contribute to conformational instability.. J Mol Biol.

[95] Martin MM, Condotta SA, Fenn J, Olmstead AD, Jean F (2011). In-cell selectivity profiling of membrane-anchored and replicase-associated hepatitis C virus NS3-4A protease reveals a common, stringent substrate recognition profile.. Biol Chem.

[96] Condotta SA, Martin MM, Boutin M, Jean F (2010). Detection and in-cell selectivity profiling of the full-length West Nile virus NS2B/NS3 serine protease using membrane-anchored fluorescent substrates.. Biol Chem.

[97] Martin MM, Jean F (2006). Single-cell resolution imaging of membrane-anchored hepatitis C virus NS3/4A protease activity.. Biol Chem.

[98] Hamill P, Hudson D, Kao RY, Chow P, Raj M (2006). Development of a red-shifted fluorescence-based assay for SARS-coronavirus 3CL protease: identification of a novel class of anti-SARS agents from the tropical marine sponge Axinella corrugata.. Biol Chem.

[99] Elagoz A, Benjannet S, Mammarbassi A, Wickham L, Seidah NG (2002). Biosynthesis and cellular trafficking of the convertase SKI-1/S1P: ectodomain shedding requires SKI-1 activity.. J Biol Chem.

[100] Seidah NG, Mowla SJ, Hamelin J, Mamarbachi AM, Benjannet S (1999). Mammalian subtilisin/kexin isozyme SKI-1: A widely expressed proprotein convertase with a unique cleavage specificity and cellular localization.. Proc Natl Acad Sci U S A.

[101] Wang X, Briggs MR, Hua X, Yokoyama C, Goldstein JL (1993). Nuclear protein that binds sterol regulatory element of low density lipoprotein receptor promoter. II. Purification and characterization.. J Biol Chem.

