# Supplementary figures and images for: Human Subtilase SKI-1/S1P Is a Master Regulator of the HCV Lifecycle and a Potential Host Cell Target for Developing Indirect-Acting Antiviral Agents
Source: PLoS Pathog. 2012 Jan 5;8(1):e1002468. doi: 10.1371/journal.ppat.1002468 (PMC3252376; doi:10.1371/journal.ppat.1002468)

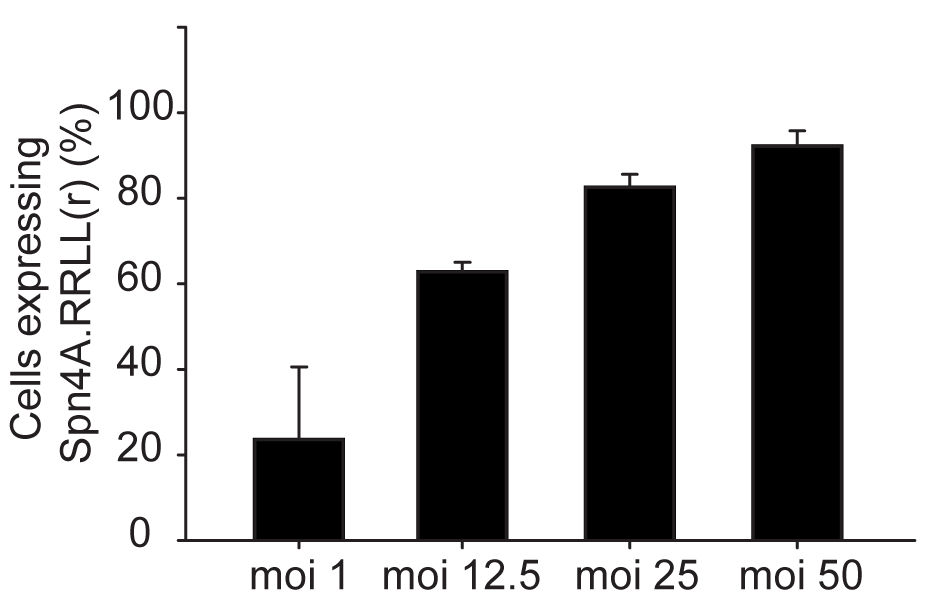

Supplement: Figure S1 — Optimization of adenovirus-expressed Spn4A.RRLL(r) expression in Huh-7.5.1 cells. Huh-7.5.1 cells were infected with moi 1, 12.5, 25, and 50 of the intracellularly retained serpin Ad-Spn4A.RRLL(r). Treated cells were fixed 48 hours post-infection and probed for serpin expression using mouse anti-FLAG antibody. Cell nuclei were stained with Hoechst dye to determine the total cell number. The percentage of Spn4A.RRLL(r)-expressing cells was quantified using Cellomics HCS. Results (mean ± SEM) from 2 independent experiments are shown. (TIF) [file ppat.1002468.s001.tif]

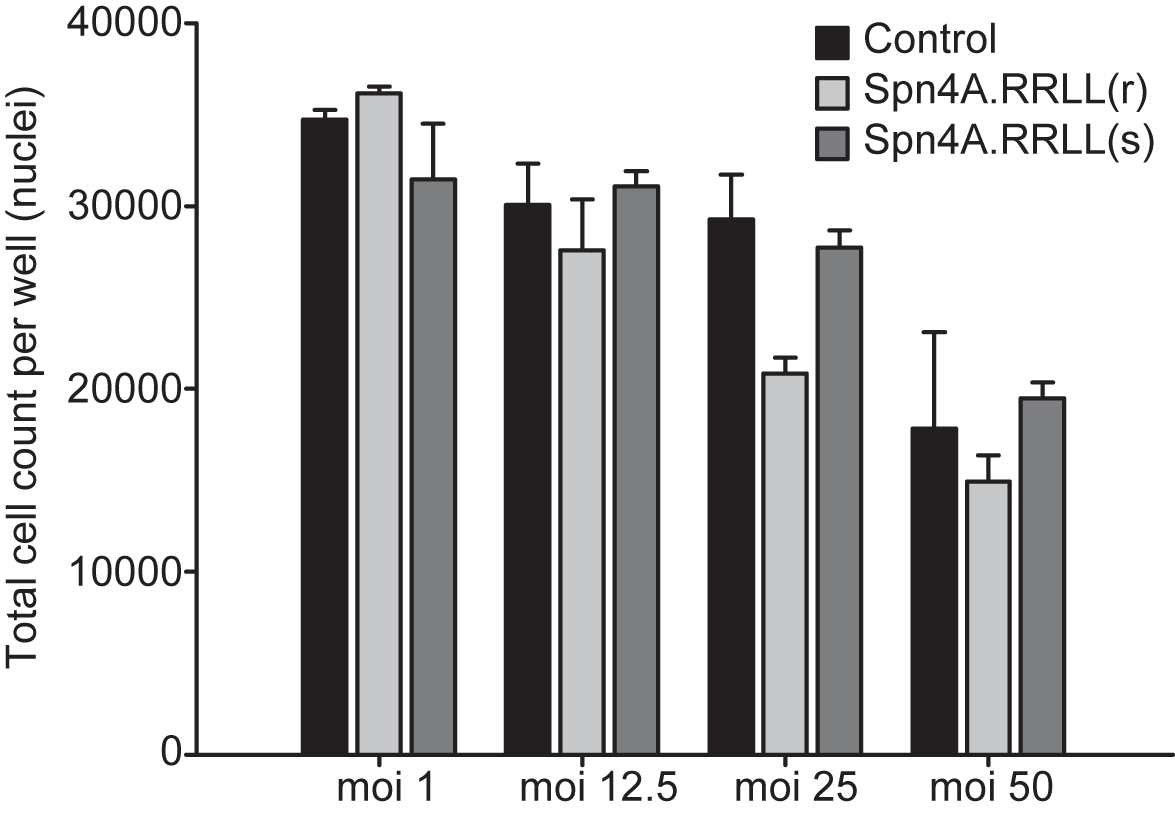

Supplement: Figure S2 — Effect of Spn4A variant treatment and HCV infection on Huh-7.5.1 cell growth. Huh-7.5.1 cells were infected with various moi (1 – 50) of Ad-Empty, Ad-Spn4A.RRLL(r), or Ad-Spn4A.RRLL(s) for 48 hours in complete media. Treated cells were infected with HCV (moi 0.1) and fixed 72 hours post-infection. Fixed cells were probed with Hoechst dye to stain for cell nuclei, which were then quantified using Cellomics HCS to determine the relative number of cells in each well under the varying conditions. All values are expressed as relative cell number in serpin-treated cells compared to cells infected with Ad-Empty, which is set to 1. Results (mean ± SEM) from 3 independent experiments are shown. (TIF) [file ppat.1002468.s002.tif]

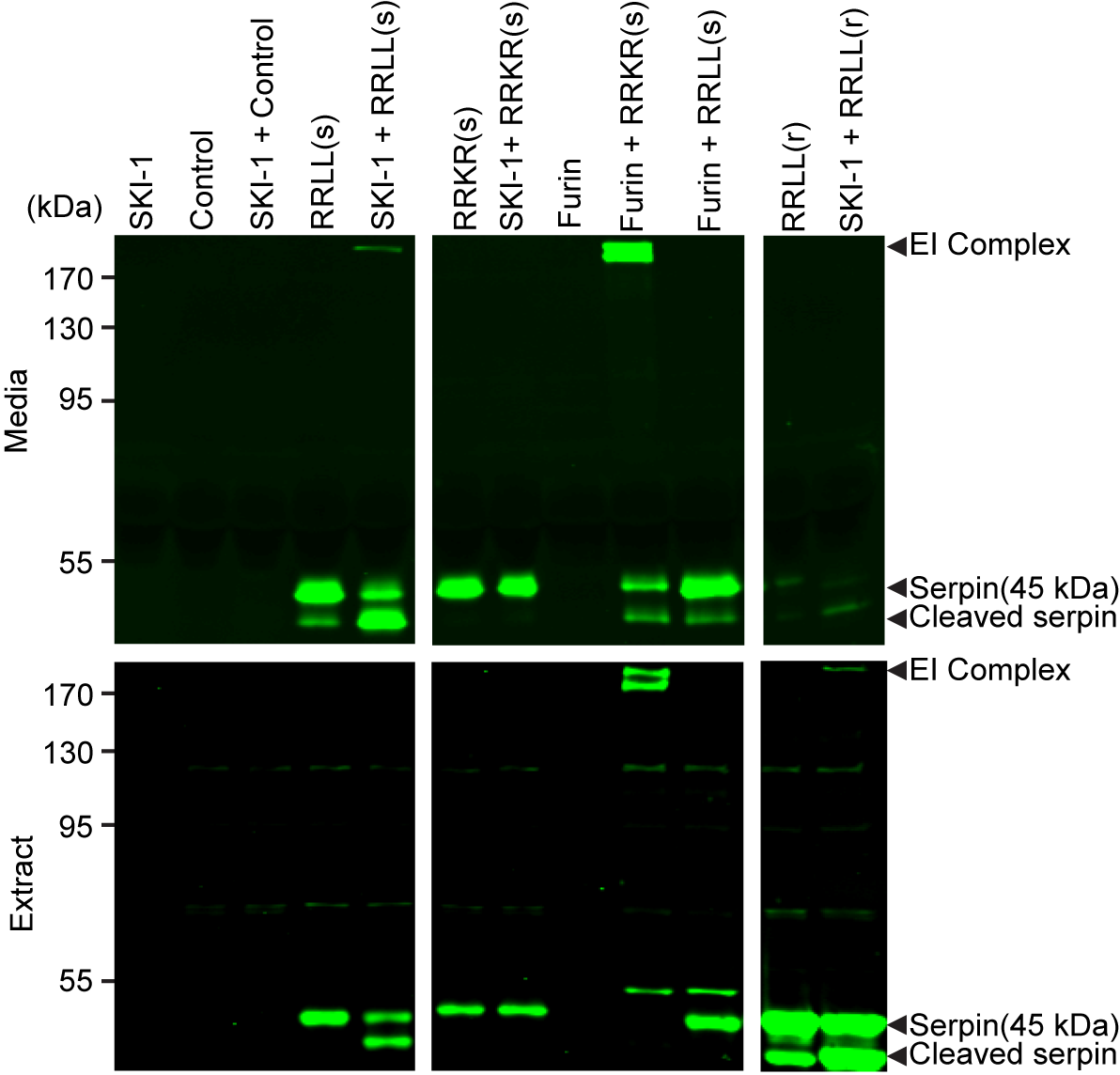

Supplement: Figure S3 — Serpin-like properties of recombinant adenovirus-expressed Spn4A variants expressed in Huh-7.5.1 cells. Huh-7.5.1 cells were infected with recombinant adenovirus expressing the His- and FLAG-tagged Spn4A variants indicated or the Ad-Empty control for 72 hours. Media alone (upper panels) or cell extracts (lower panels) lysed in RIPA buffer were combined with recombinant His-tagged SKI-1/S1P [47] or His-tagged furin for 30 minutes at 30°C. Samples were prepared for Western blot analysis and probed with rabbit anti-FLAG antibody to detect SDS- and heat-stable protease-serpin complex formation and also to distinguish serpin bands from protease bands on the Western blots. All Western blots shown are representative of at least 2 independent experiments. (TIF) [file ppat.1002468.s003.tif]

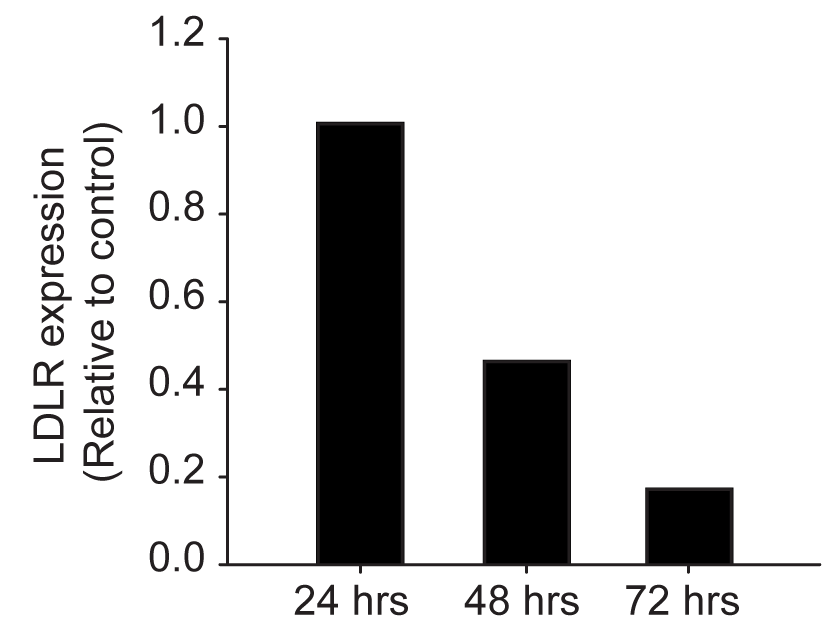

Supplement: Figure S4 — Time course analysis of LDLR expression in Spn4A.RRLL(s)-treated cells. Huh-7.5.1 cells were grown in complete media for 24 hours and then infected with Ad-Empty (control) or Ad-Spn4A.RRLL(s). Cell extracts were harvested for Western blot 24, 48, and 72 hours post-infection, and lysates were then subjected to Western blot. Anti-LDLR antibody was used to detect protein-expression levels in control- and Ad-Spn4A.RRLL(s)-treated cells, and β-tubulin was probed for normalizing LDLR expression. Values are plotted relative to LDLR expression in control (Ad-Empty)-treated cells, which was set to 1. The representative results of 2 independent experiments are shown. (TIF) [file ppat.1002468.s004.tif]

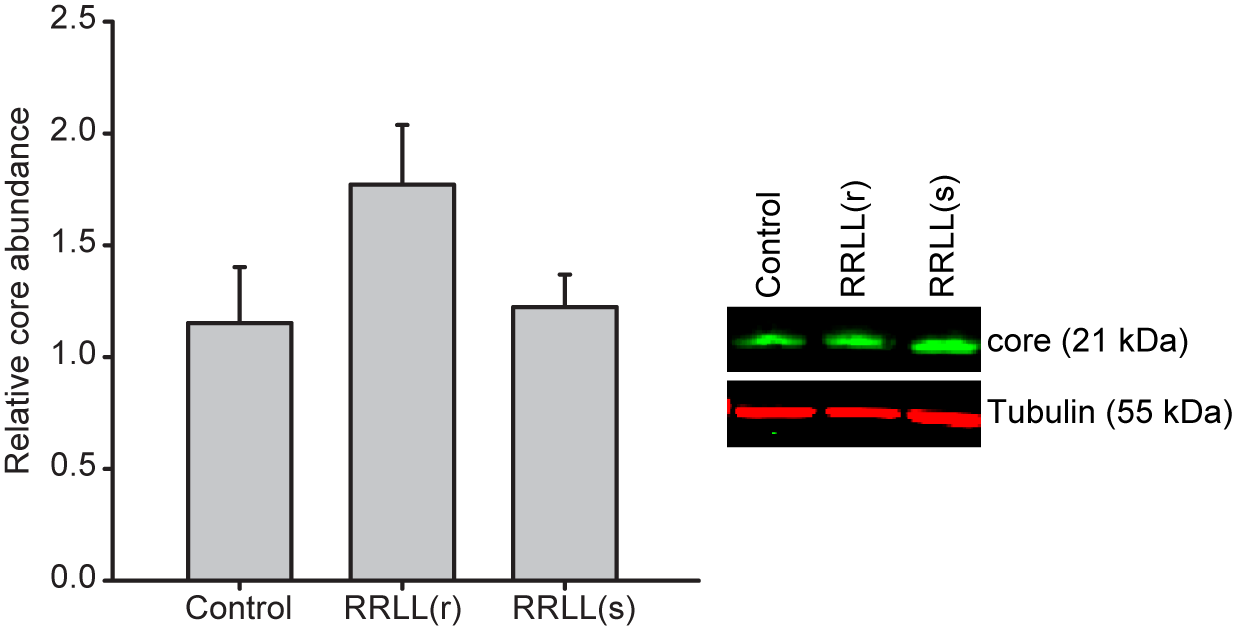

Supplement: Figure S5 — Spn4A.RRLL(s) does not block HCV core production post-transfection of HCV RNA. Huh-7.5.1 cells were infected with Ad-Empty (control), Ad-Spn4A.RRLL(r), or Ad-Spn4A.RRLL(s) (moi 50) for 48 hours in complete media and then transfected with genomic HCV RNA for 72 hours. Relative HCV-core expression (normalized to β-tubulin) in serpin-treated cells compared to control-treated cells was quantified by examining total cell lysates using Western blot analysis. Results (mean ± SEM) from 2 independent experiments are shown. A representative Western blot is shown to the right of the graph. (TIF) [file ppat.1002468.s005.tif]

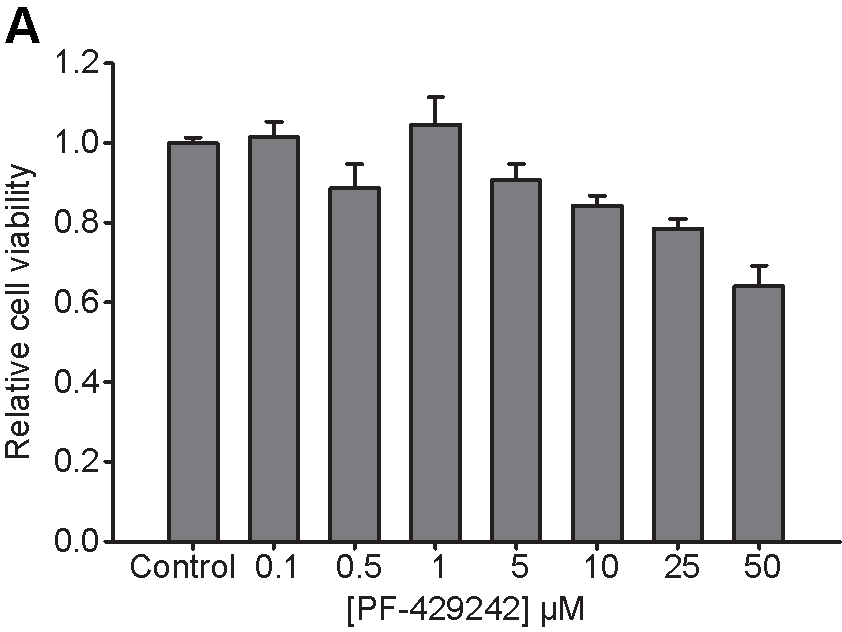

Supplement: Figure S6 — The effect of PF-429242 on cell viability. Huh-7.5.1 cells were treated with DMSO (control) or various concentrations of PF-429242 for 24 hours before the inhibitor was removed, and fresh complete media was added to the cells for an additional 48 hours. The relative cytotoxicity of the compound was then determined using an MTS-based cell viability assay. The absorbance measured at 490 nm is proportional to the number of living cultured cells. Results (mean ± SEM) from 3 independent experiments are shown. Statistical significance was calculated for PF-429242-treated cells compared to DMSO-treated cells. (TIF) [file ppat.1002468.s006.tif]
